# Extensive cryptic circulation sustains mpox among men who have sex with men

**DOI:** 10.1038/s41467-026-72749-2

**Published:** 2026-05-13

**Authors:** Joseph A. Lewnard, Miguel I. Paredes, Matan Yechezkel, Gregg S. Davis, Vennis Hong, Jessica Skela, Utsav Pandey, Noah T. Parker, Lauren C. Granskog, Magdalena E. Pomichowski, Iris Anne C. Reyes, Isabel Rodriguez-Barraquer, Nicola F. Müller, Sara Y. Tartof

**Affiliations:** 1 Division of Epidemiology, School of Public Health, University of California, Berkeley, Berkeley, CA USA; 2 Center for Computational Biology, College of Computing, Data Science, and Society, University of California, Berkeley, Berkeley, CA USA; 3 Vaccine and Infectious Disease Division, Fred Hutchinson Cancer Center, Seattle, WA USA; 4 Department of Research & Evaluation, Kaiser Permanente Southern California, Pasadena, CA USA; 5 Regional Reference Laboratories, Kaiser Permanente Southern California, Chino Hills, CA USA; 6 Department of Clinical Science, Bernard J. Tyson School of Medicine, Pasadena, CA USA; 7 Department of Epidemiology, Johns Hopkins Bloomberg School of Public Health, Baltimore, MD USA; 8 Division of HIV, Infectious Diseases, and Global Medicine, Department of Medicine, University of California, San Francisco, San Francisco, CA USA; 9 Chan Zuckerberg Biohub, San Francisco, CA USA; 10 Department of Health Systems Science, Kaiser Permanente Bernard J. Tyson School of Medicine, Pasadena, CA USA

**Keywords:** Epidemiology, Viral infection, Risk factors

## Abstract

Sporadic cases of clade IIb mpox continue to be notified among men who have sex with men, with most lacking identifiable transmission links. To resolve underlying dynamics, we tested prospectively for evidence of monkeypox virus infection in anorectal swabs from a cohort of men who have sex with men in Los Angeles, whom we monitored concurrently for clinical mpox diagnoses during summer, 2024. Here we estimate that infections exceeded reported mpox cases by a 33-fold margin (95% confidence interval: 16-68). Independent estimates derived from phylogenetic reconstruction and a meta-analysis of surveillance studies corroborated this extensive under-reporting. We estimate that undiagnosed infections must cause at least 31-44% of all transmission to explain observed monkeypox virus phylogenies; under realistic modeling assumptions, this proportion rises to 61-97%. Contrary to current guidance, our findings suggest a substantial proportion of monkeypox virus circulation may be associated with subclinical infections, challenging the feasibility of current mpox elimination targets.

## Abstract (web summary)

The authors analysed a cohort of men who have sex with men for diagnosed and asymptomatic mpox and report that only 1 in 33 infections is captured by clinical testing. The results indicate that there may be extensive under-reporting and under-diagnosis of mpox.

## Introduction

Since 2022, clade IIb monkeypox virus (MPXV) has spread sexually among men who have sex with men (MSM) and other sexual- and gender-minority populations in countries where it was previously non-endemic^1^. Clinically, mpox is associated with a prodrome characterized by fever, chills, myalgia, and lymphadenopathy, followed by painful cutaneous or mucosal lesions, often localized to sites of sexual exposure^2^. Current guidance from the US Centers for Disease Control & Prevention (CDC) and European Centre for Disease Prevention & Control (ECDC) stipulates that subclinical or asymptomatic infections are uncommon^3,4^. Accordingly, declines in reported case counts to sporadic detections in most countries have been interpreted as evidence of successful containment^5^, with ECDC and the World Health Organization (WHO) targeting elimination of human-to-human transmission by 2027 in countries with outbreaks driven by sexual transmission^6,7^.

However, several observations have suggested that cryptic transmission may undermine this objective. Although numerous health agencies recommend contact tracing as a strategy to interrupt transmission^8^^–^^11^ >90% of mpox cases have lacked epidemiological links to prior cases, and 67–85% have reported no known exposure to individuals with mpox or mpox-like symptoms^2,12–15^. Observations that individuals shed replication-competent MPXV before symptoms onset and during subclinical infection^16–18^, and widely documented examples of pre-symptomatic transmission^2,19–21^, suggest contact with lesions is not required for MPXV transmission. Quantifying the proportion of individuals that become symptomatic and receive mpox diagnoses could clarify the epidemiological importance of subclinical MPXV infections. However, ascertainment ratios for clade IIb MPXV remain unknown.

To resolve this uncertainty, we launched a prospective surveillance study among Los Angeles MSM receiving healthcare from Kaiser Permanente Southern California (KPSC). The KPSC healthcare system provides comprehensive, integrated care to members; electronic health records (EHRs) record all diagnoses, clinical notes, laboratory tests, procedures, immunizations, and pharmacy prescription fills. Insurance reimbursement data capture out-of-network care, and history of vaccination with JYNNEOS (modified *Vaccinia* Ankara) is automatically reconciled with the California Immunization Registry, to which providers statewide are required to report all vaccine administrations. Our study cohort comprised 7930 males aged 16–52 years on May 1, 2024, with ≥1 year of prior enrollment and ≥1 anorectal test for *Chlamydia trachomatis* or *Neisseria gonorrhoeae* infection (as receptive anal sex exposures are the basis this testing indication, our inclusion criteria restricted the study population to MSM). From May 29 to November 13, 2024, we tested eligible cohort members’ remnant anorectal specimens collected for *C. trachomatis*/*N. gonorrhoeae* screening—which sexually-active MSM are recommended to receive as often as every 3–6 months—for MPXV DNA via real-time polymerase chain reaction (PCR), while concurrently monitoring for mpox diagnoses in any clinical care setting.

## Results

### Subclinical MPXV infections vastly outnumber diagnosed cases

**Fig. 1:**
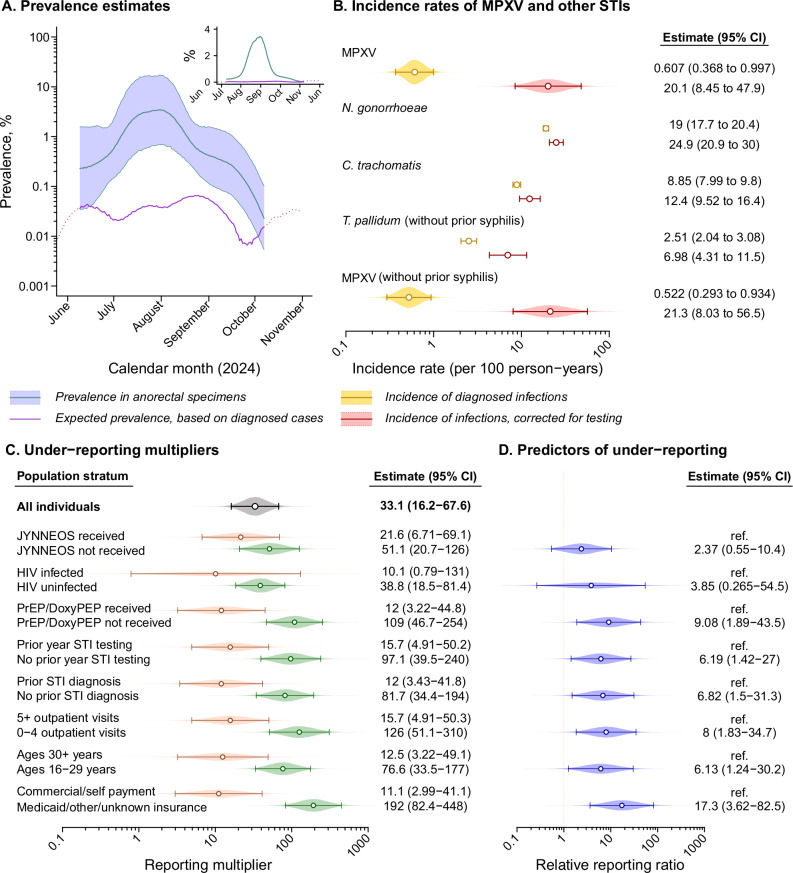
Prevalence and incidence of MPXV infection, and predictors of under-reporting. **A** We present estimates of expected prevalence of MPXV infection accounting only for shedding among cases receiving mpox diagnoses (purple line) alongside estimates of observed prevalence based on anorectal specimen testing (blue line, shaded area corresponding to 95% confidence intervals, presented as a moving 3-week average). Dotted lines convey prevalence estimates outside the period where a 3-week moving average could be computed for MPXV detection in anorectal specimens. We illustrate results on an absolute scale in the top-right inset panel. Estimates are weighted to adjust for differences in characteristics of individuals for whom anorectal specimens were collected or not collected during the study period. We detail c_*T*_ values for MPXV-specific and NVAR probes in Table S3. **B** Incidence rates of sexually-transmitted infections based on diagnosed cases (blue) and estimated rates of infection, corrected for testing effort (red). Rates are presented as infections per 100 person-years during the study period, within the weighted study cohort, for MPXV, *Neisseria gonorrhoeae*, *Chlamydia trachomatis*, and *Treponema pallidum*. Rates for *T. pallidum* are presented for individuals with no history of prior syphilis, due to use of serological diagnosis; rates for MPXV among individuals without prior syphilis diagnoses are presented below. Points convey maximum likelihood estimates, and lines convey 95% confidence intervals. **C** Estimates of the reporting multiplier, overall and for subgroups defined by demographic and clinical characteristics. We present alternative estimates based on an instantaneous prevalence comparison in Table S4 along with results of sensitivity analyses excluding repeat positives or closely-spaced sampling intervals, and results based on alternative parameterizations of the duration of shedding in Table S5. We present results adjusted for the association of positive PCR detection with culture-confirmed MPXV shedding and anti-MPXV IgG seroconversion in Table S8. Points convey maximum likelihood estimates, and lines convey 95% confidence intervals. **D** Ratios comparing the estimated reporting multiplier across the distinct subgroups for which we present data. Points convey maximum likelihood estimates, and lines convey 95% confidence intervals. Estimates throughout **A**–**D** are derived from analyses including 7930 individuals followed for mpox diagnoses via electronic health records, among whom 1054 received tests for subclinical MPXV infection via anorectal specimens.

The incidence rate of laboratory-confirmed mpox diagnoses identified through standard-of-care testing in clinical settings was 0.61 (95% confidence interval: 0.27–0.77) cases per 100 person-years at risk among cohort members (*n* = 15 cases, among 53 individuals who received diagnostic testing for mpox) in analyses that balanced characteristics of individuals who received or did not receive screening tests through inverse propensity weighting based on their demographics, healthcare utilization, and history of sexually-transmitted infection (STI) diagnoses (Figure S1; Table S1). Accounting solely for infections among diagnosed cases, we expected the weighted prevalence PCR-positive detections to be 0.035% in anorectal swabs (Fig. 1A, Table S2, and Fig. S2). In contrast, testing 1190 remnant anorectal specimens from 1054 individuals, we identified 7 positive results among 6 individuals, yielding markedly higher estimates of weighted prevalence (0.91% [0.40–1.6%] and incidence (20 [8.5–48] infections per 100 person-years; see “Methods” for description of testing procedures). All positive specimens had cycle threshold (c_*T*_) values below the validated detection limit for both an MPXV-specific probe and a non-Variola *Orthopoxvirus* (NVAR) probe. Within each specimen, c_*T*_ values were similar for the MPXV-specific and NVAR probes (range in absolute differences: 0.5–0.9; Table S3). This alignment in quantitative results was closer than expected by chance among positive specimens (two-sided *p* < 0.0001 for bootstrapped permutation tests), suggesting detections were not spurious results of either assay.

No individuals testing PCR-positive for MPXV via anorectal specimens sought clinical care relating to mpox symptoms or received diagnostic testing during the study period, suggesting these detections were associated with asymptomatic or paucisymptomatic infections. In most respects, characteristics of the 6 individuals with MXPV detected via anorectal specimen surveillance closely resembled characteristics of those receiving mpox diagnoses (Table S2); one was receiving treatment for HIV infection, while 4 were receiving tenofovir-emtricitabine for HIV pre-exposure prophylaxis. The remaining individual not receiving antiviral treatment for HIV care or prevention was tested twice during the study period for STIs, and received antibiotic treatment for both *N. gonorrhoeae* and *C. trachomatis* concurrently with his positive MPXV swab.

We next used a likelihood-based framework to estimate a reporting multiplier relating daily anorectal MPXV test positivity to expected rates of infection acquisition, shedding, and clearance derived from diagnosed cases^1,17,22^. By this approach, we estimated that only 1 in 33 (16–68) MPXV infections received a laboratory-confirmed mpox diagnosis (Fig. 1C). Reporting multiplier estimates varied by individuals’ healthcare engagement and demographic characteristics; predictors of lower reporting included younger age, lack of HIV pre-exposure prophylaxis or doxycycline post-exposure prophylaxis, no prior-year STI testing or diagnoses, having <5 healthcare visits in the prior year, and receipt of health insurance through non-commercial sources (Fig. 1D).

Analyses excluding repeat positive results or closely-spaced consecutive swabs (<30 days) yielded similar results, while an alternative, cross-sectional analysis comparing time-matched observed and expected MPXV prevalence estimated that 1 in 26 (12–47) infections was diagnosed (Table S4). Point estimates from analyses using alternative data sources to parameterize the expected duration of PCR-positive anorectal MPXV shedding^23,24^ fell within the 95% confidence interval of our primary estimates (1 in 21 [11–43] to 1 in 52 [25–106]; Table S5).

Because our study could be susceptible to bias if PCR-positive anorectal specimens from individuals without clinical illness included false-positive detections, we also undertook several analyses addressing the specificity of this under-reporting signal. Across 4 prospective studies enrolling participants under symptoms-agnostic frameworks and attempting viral culture^16–18,25^, specimens from 8 out of 11 (73%) individuals with PCR-positive anorectal specimens yielded replicating MPXV (Table S6). Associations of c_*T*_ value with successful MPXV culture from anorectal specimens were equivalent among individuals with subclinical detections and those with clinical illness^26^ (*p* ≥ 0.4 across tests for differences in slopes; Table S7 and Fig. S3). Analyses accounting for the duration of culturable MPXV shedding—and for the probability of recovering culturable MPXV at c_*T*_ values observed in our study—yielded a reporting multiplier of 1 in 39 (17–90) infections, closely resembling our primary estimate (Table S8). Analyses accounting for individuals’ probability of anti-MPXV IgG seroconversion after subclinical PCR-positive MPXV detection (reported in 8 of 9 individuals across prior studies collecting follow-up sera^16,17^) yielded an adjusted reporting multiplier of 1 in 31 (14–62) infections (Tables S6 and S8). Our estimates also held when considering test specificity well below previously-published estimates^27^, with risk of false positive results mitigated by our use of two distinct probes in qPCR assays (Fig. S4).

Contrary to the low apparent incidence of mpox diagnoses, the estimated incidence rate of 20 (8.5–48) MPXV infections per 100 person-years within the KPSC study cohort was comparable to bacterial STIs for which MSM are recommended to receive routine testing. Estimated incidence rates of *N. gonorrhoeae* and *C. trachomatis* infection totaled 25 (21–30) and 12 (9.5–16) acquisitions per 100 person-years, respectively, over the same period (Fig. 1B). Among persons without prior syphilis diagnoses, incidence of syphilis (*Treponema pallidum*) infections was substantially lower than incidence of MPXV infection (7.0 [4.3–11.5] versus 21 [8.0–57] acquisitions per 100 person-years), further highlighting the unexpected scale of MPXV transmission.

### Vaccination and MPXV infection

**Fig. 2:**
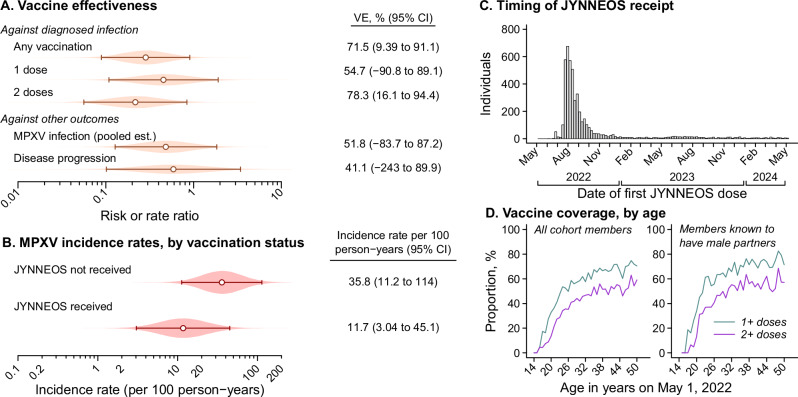
Testing-corrected incidence rates and vaccine effectiveness estimates. **A** Estimates of JYNNEOS vaccine effectiveness against diagnosed mpox, against MPXV infection, and against progression to diagnosed mpox, given MPXV infection. Effect estimates against diagnosed mpox are based on a case-control analysis comparing vaccination status among diagnosed mpox cases to individuals diagnosed with gonorrhea. Estimates of effectiveness against MPXV infection are pooled across analyses based on the incidence rate ratio of MPXV infection comparing vaccinated to unvaccinated individuals, and comparing prior vaccination among individuals testing positive or negative for MPXV infection by anorectal swabbing; estimates of effectiveness against disease progression are derived mathematically from estimated effectiveness against diagnosed mpox and against MPXV infection (Table S9). Points convey maximum likelihood estimates, and lines convey 95% confidence intervals **B** Incidence rates of MPXV infection, corrected for testing effort, stratified by individuals’ history of JYNNEOS vaccination. Points convey maximum likelihood estimates and lines convey 95% confidence intervals. Estimates plotted in **A**, **B** are derived from analyses including 7930 individuals followed for mpox diagnoses via electronic health records, among whom 1054 received tests for subclinical MPXV infection via anorectal specimens (**C**) Dates of receipt of first JYNNEOS doses among vaccinated cohort members. (**D**) Coverage of ≥1 or ≥2 JYNNEOS doses among cohort members as of May 1, 2024, plotted against an axis indicating individuals’ age as of 1 May, 2022. Panels include all 7930 cohort members (left) as well as those with clinical notes indicating provider awareness of male sex partners (right).

Among the study cohort, pre-exposure vaccination with JYNNEOS was associated with 72% (9.4–91%) effectiveness against diagnosed mpox (78% for two doses, 55% for one dose; Fig. 2A and Table S9), consistent with prior estimates^28^. Although inconclusive due to the low number of infections observed, our observation that previously vaccinated individuals accounted for 5 (83%) of the 6 subclinical infections identified through anorectal specimen testing, but 7 (47%) of the 15 cases receiving mpox diagnoses, aligned with prior evidence that vaccination with JYNNEOS may protect against mpox, in part, by reducing disease severity and the likelihood of diagnosed illness^29–31^. Weighted incidence rates of MPXV infection were 51% (−92 to 95%) lower among JYNNEOS recipients than non-recipients (Fig. 2B), and vaccination was associated with 53% (−167 to 92%) effectiveness against anorectal MPXV infection within our surveillance study (59% for two doses, 41% for one dose; two-sided *p* > 0.1 for both comparisons).

Nearly all vaccinated individuals within the study population received JYNNEOS during acute phases of the mpox outbreak between July and October, 2022 (Fig. 2C) amid expanded JYNNEOS delivery efforts^32^. Low coverage among individuals aged <18 years in 2022 (12%) in comparison to individuals aged ≥18 years (60%) demonstrates ongoing growth of an undervaccinated cohort amid continued MPXV circulation (Fig. 2D).

### Implications of subclinical infections for MPXV circulation

While these findings suggested that MPXV infections outnumber diagnosed cases, the contribution of subclinical or undiagnosed infections to MPXV transmission remains uncertain^3,4,33^. To understand whether mpox epidemiologic observations are consistent with scenarios where individuals with undiagnosed infection contribute or do not contribute to onward spread, we next modeled MPXV transmission, accounting for dispersion in individuals’ number of secondary infections via a negative binomial offspring distribution (see “Methods”). Under a scenario in which undetected MPXV infections do not transmit infection^3,25,34,35^, diagnosed mpox cases (representing 3.0% of total infections) would necessarily account for all onward spread. Such extreme concentration of transmission implies superspreading dynamics characterized by a very low dispersion parameter (*k*), which quantifies heterogeneity in infectiousness in epidemiological models^36–38^.

**Fig. 3:**
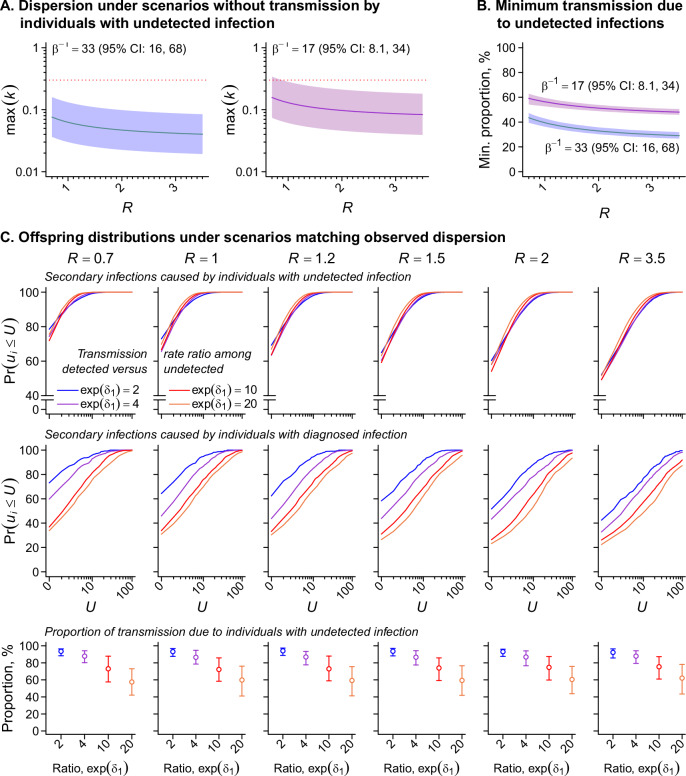
Modeling the contribution of individuals with diagnosed mpox and undetected MPXV infection to transmission. **A** Maximum values of the dispersion parameter, *k*, obtained under scenarios where only individuals with detected infections transmit MPXV. Modeled scenarios minimize dispersion in the offspring distribution by assuming uniform numbers of secondary infections for the proportion $(1-\beta )$ of individuals with diagnosed mpox, and no secondary infections for the proportion $\beta$ of individuals with undetected MPXV infection. The reproduction number *R* denotes the mean number of secondary infections caused by each infected individual. We consider scenarios with reporting multipliers of ${\beta }^{-1}=33$ (per our primary analysis) and ${\beta }^{-1}=17$ (as a sensitivity analysis). Shaded areas delineate 95% confidence intervals around our estimates, and dotted red lines correspond to $k=0.3$, as estimated in previous studies^39–41^. **B** Minimum proportion of transmission attributable to undetected infections, under scenarios with $k=0.3$ and deterministic assignment of transmission links to index cases with diagnosed mpox. We present estimates with reporting multipliers of ${\beta }^{-1}=33$ (per our primary analysis) and ${\beta }^{-1}=17$, as a sensitivity analysis. **C** Offspring distribution characteristics under scenarios with differing transmission dynamics. Top and middle rows present cumulative distribution functions for the number of secondary infections (*U*) caused by individuals with undetected MPXV infection and diagnosed mpox, respectively, varying the rate ratio of transmission by individuals with diagnosed mpox relative to those with undetected MPXV infection, $\exp ({\delta }_{1})$, from 2 to 20. Bottom row panels indicate the total proportion of transmission attributable to individuals with undetected infection for each value of ${\delta }_{1}$. Panels are grouped into columns according to *R* values employed for each analysis (*R* = 0.7, 1.0, 1.2, 1.5, 2.0, 3.5). Lines illustrate 95% confidence intervals surrounding point estimate (circles). Estimates throughout **A**–**C** are derived from analyses including 7930 individuals followed for mpox diagnoses via electronic health records, among whom 1054 received tests for subclinical MPXV infection via anorectal specimens.

However, contrary to this scenario, phylogenetic analyses have estimated *k* values between 0.30 and 0.32 for MPXV transmission during the current clade IIb outbreak^39–41^, similar to estimates for *N. gonorrhoeae* among MSM (*k* = 0.26)^42^. Models excluding transmission by undetected infections—and minimizing dispersion by assuming uniform numbers of secondary infections among all diagnosed mpox cases—yielded maximal *k* estimates well below these previous estimates, ranging from 0.047 (0.021–0.092) to 0.082 (0.036–0.16) for scenarios with a reproduction number (*R*) between 0.7 and 3.5 (Fig. 3A). Sensitivity analyses allowing transmission by 6.0% of the population—consistent with a higher reporting multiplier or 50% ascertainment fraction for individuals with clinical illness—likewise provided weak statistical support for observed patterns (2.3-11% probability for *k* ≥ 0.3 with *R* < 1, and <1% probability with *R* ≥ 1).

We therefore quantified the contribution of undetected infections to transmission under more realistic scenarios with MPXV dispersion characteristics mirroring prior observations (*k* = 0.3). Maximizing the proportion of transmission attributable to diagnosed cases via a deterministic framework, we estimated that individuals with undetected infection must account for a minimum of 31% (29–34%) to 44% (40–47%) of all transmission events (Fig. 3B). This estimated attributable fraction exceeded half of all transmission under less-restrictive modeling assumptions allowing differences in infectiousness among individuals with diagnosed or undiagnosed infections (Fig. 3C). Assuming individuals with diagnosed infection are 20-fold more likely to transmit MPXV compared to undetected cases, the proportion of transmission attributable to undetected infections was 61% (43–78%). Assuming weaker differences in infectiousness (only a 2-fold increase for diagnosed cases relative to undetected infections), the contribution of undetected infections rose to 94% (88–97%), collapsing to the proportion of infections that go undetected (97% [94–99%]) when assuming no difference in infectiousness among detected and undetected infections (Table S7). These findings suggest undetected infections must play a meaningful role in sustaining viral transmission to explain dispersion patterns evident in MPXV phylogenies.

**Fig. 4:**
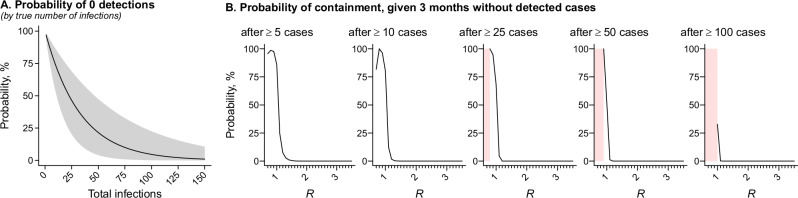
Elimination thresholds for mpox. **A** Probability of observing 0 diagnosed mpox cases, based on true number of infections, assuming 3.0% (1.5–6.2%) of infections receive diagnoses. The shaded area delineates 95% confidence bounds around maximum likelihood estimates (lines). We generate estimates assuming the number of diagnosed cases is a binomial random variable. **B** Probability of containment, defined as the cessation of ongoing transmission 90 days after the last notified case. Panels illustrate the estimated probability of containment, generated via simulation of transmission chains with a negative-binomial offspring distribution and varying values of *R*, under scenarios where ≥5, ≥10, ≥25, ≥50, and ≥100 notified cases occurred prior to the last notified case. Shaded pink areas delineate ranges of *R* where all simulated transmission chains were depleted before the minimum number of notified cases occurred.

Current guidance defines elimination of human-to-human transmission as the absence of new, locally-acquired mpox cases (without travel history or zoonotic exposure) for ≥3 months in settings with adequate capacity to conduct laboratory testing for individuals meeting clinical case criteria^8^. However, at our estimated reporting multiplier, the probability of observing 0 cases would exceed 50% under scenarios where up to 23 (11–47) infections had occurred, and would exceed 5% with as many as 98 (48–202) true infections (Fig. 4A). In modeling analyses considering our estimated reporting fraction of 1 in 33 infections, presence of 0 diagnosed cases over a 3-month span was unlikely to indicate absence of ongoing transmission under most scenarios with *R* ≥ 1 (Fig. 4B). With *R* = 1.1, the probability of containment after 3 months without detected cases was 25%, 13%, 4.5%, 1.2%, and 0.14% in scenarios where this lapse in detections was preceded by outbreaks involving 5, 10, 25, 50, and 100 or more notified cases, respectively. The same probabilities were <10% for scenarios with *R* ≥ 1.2, and <1% for scenarios with *R* ≥ 1.5. Because undetected MPXV circulation may persist over extended periods without clinically-detected cases, expanded surveillance efforts, including asymptomatic testing in high-risk populations, may offer an important strategy for monitoring transmission.

### Under-reporting is geographically widespread and confirmed by phylogenetic analyses

To understand the external generalizability of our results, we next compared evidence of under-reporting within the KPSC cohort to data from other settings. Numerous surveillance studies have identified undetected MPXV infections based on PCR-positive anorectal shedding or serological evidence of prior infection, although implications of these findings for local mpox reporting fractions remain unknown. We undertook a systematic review of epidemiological studies conducting molecular or antibody-based testing for MPXV infection among MSM, and compared findings from 10 studies meeting eligibility criteria^16,18,25,35,43–48^ to mpox case notifications from the same settings (Tables S10–S12 and Fig. S4).

**Fig. 5:**
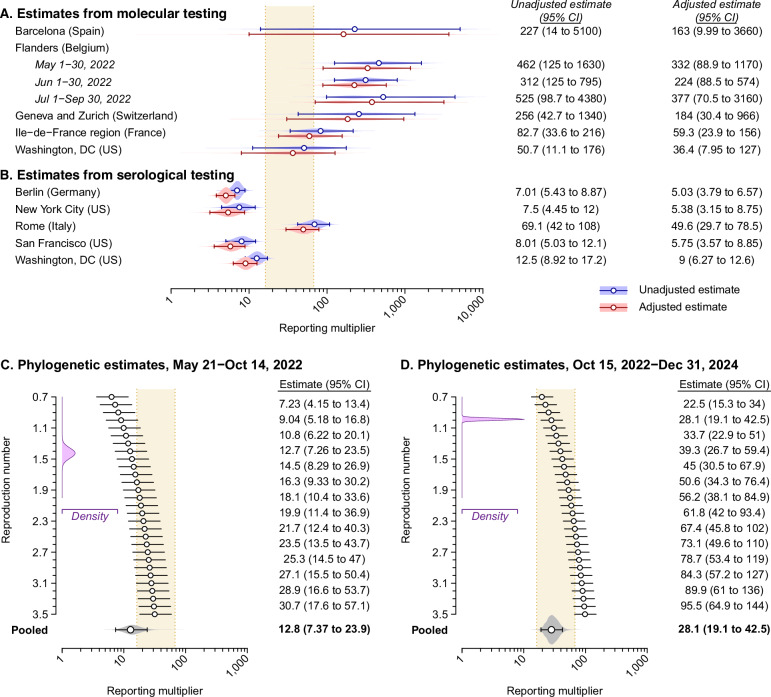
Reporting multiplier estimates based on external surveillance studies and phylogenetic analyses. **A** Estimated reporting multipliers comparing MPXV infection prevalence observed in anorectal specimen testing to expected prevalence based only on diagnosed cases in each study setting. We detail the included studies and data providing the basis for estimates in Tables S10 and S11. Risk-adjusted estimates account for expected differences in MPXV detection in samples recruited in STI clinic settings as compared to the general MSM population (Fig. S6). The tan shaded area delineates the 95% confidence interval around our primary estimate within the KPSC study population (33; 95% confidence interval [CI]: 16–68) as a basis for comparison. Studies included, in total, 1228 individuals tested via anorectal specimens. **B** Estimated reporting multipliers comparing anti-MPXV IgG seroprevalence to expected seroprevalence based on cumulative diagnosed cases in the study setting. We present pooled estimates of reporting multipliers across studies, with and without risk adjustment, in Table S12. Studies included, in total, 1764 individuals tested for serological evidence of prior infection. **C** Reporting multipliers based on a comparison of diagnosed cases to the estimated population size of MPXV infections in Los Angeles County from phylogenetic analyses of sequenced cases, for the period from May 21 to October 14, 2022. Estimates are conditioned on the reproduction number, *R*. We illustrate the probability density of mean daily *R* values throughout the analysis period in purple (top left inset panel). Pooled estimates presented in bold at the bottom of the figure are weighted according to the period-specific probability density of *R*. We present alternative estimates assuming lower or higher values of the dispersion parameter *k* in Fig. S8. Analyses include 271 sequenced Los Angeles County mpox cases. **D** Estimated reporting multipliers comparing diagnosed cases to the estimated population size of MPXV infections in Los Angeles County, based on phylogenetic analyses, for the period from October 15, 2022, to December 31, 2024. Analyses include 226 sequenced Los Angeles County mpox cases. Estimates throughout **A**–**D** are plotted as maximum likelihood estimates (points) with accompanying 95% confidence intervals (lines).

In studies testing for PCR-positive MPXV shedding via anorectal specimens^16,18,25,43–45^, observed prevalence exceeded expected prevalence based on reported cases by factors of 51–525 (Fig. 5A). Adjusting for potential higher-risk behavior in tested populations (Fig. S5), estimated reporting multipliers ranged from 1 in 36–377. Serological studies revealed a lower, but still substantial, extent of under-reporting (1 in 7.0–69 infections, unadjusted; 1 in 5.0–50, adjusted; Fig. 5B)^25,35,46–48^. In the single study employing both molecular and serological testing^25^, reporting multipliers estimated from serological testing revealed 4.0-fold (0.86–15) greater reporting completeness than estimates based on molecular testing results, suggesting differences were driven by lower sensitivity of serological testing.

To further validate our findings using a distinct analytical framework and data source, we next estimated reporting multipliers via phylogenetic reconstruction of MPXV transmission within Los Angeles County. Leveraging data from 497 local clade IIb MPXV genomes (associated with 287 unique introductions) alongside 7140 publicly available global sequences collected between May 2022 and December 2024, we fit a birth-death skyline model^49^ to local outbreak clusters using Markov chain Monte Carlo in BEAST2 (version 2.7.8), as described previously^39,40,50^, and used estimates of the sampling proportion to compute reporting multipliers^51^. Validating this framework for estimating the sampling proportion in the presence of transmission heterogeneity (Fig. S6), we estimated that MPXV infections outnumbered reported cases by a factor of 28 (19–43) from October 15, 2022, to December 31, 2024, closely resembling our primary estimate of 33 (16–68) based on anorectal specimen testing. We also estimated that MPXV infections exceeded reported cases by a factor of 13 (7.4–24) during the preceding emergency phase of the outbreak (Figs. 5C, D and S7). This 49% (23–96%) reduction in the proportion of MPXV infections receiving diagnoses after October 15, 2022, aligns with the rapid rollout of JYNNEOS for at-risk MSM: 76% of Los Angeles County recipients of JYNNEOS^52^, and half of the full KPSC study cohort, were vaccinated by October 15, 2022.

## Discussion

Our results suggest substantial incidence of undetected clade IIb MPXV infections among MSM, with infections outnumbering diagnosed cases by a 33-fold margin within a cohort of Los Angeles MSM during summer, 2024. Estimated reporting multipliers spanned 1 in 21 to 1 in 52 in analyses using alternative parameterizations of MPXV natural history characteristics, while independent datasets derived from molecular surveillance, serological studies, and phylogenetic analyses supported this finding of extensive under-reporting. In our study cohort, most subclinical infections occurred among individuals who previously received JYNNEOS, in agreement with prior evidence that vaccination may reduce disease severity^29–31^. The low estimated reporting fraction further implies that undetected infections may contribute to transmission, contradicting current guidance that suggests undetected infections are uncommon^4,33,34^ and emphasizes symptom-driven transmission^3^. Our outcomes suggest that currently-endorsed elimination thresholds based on the absence of reports of locally-acquired cases for ≥3 months may provide a poor proxy for resolution of outbreaks. Ongoing cryptic circulation of MPXV may undermine the effectiveness of currently-endorsed public health strategies focused on identifying cases with clinical illness and tracing their contacts^8–11^, and underscores the importance of renewed vaccination efforts to protect populations at high risk of exposure.

To our knowledge, no prior studies have directly estimated the proportion of MPXV infections that are clinically diagnosed based on results of symptomatic and asymptomatic testing. A study prospectively monitoring contacts of mpox cases for MPXV infection reported symptoms among 46% of individuals who tested positive^17^, although intensive monitoring likely enhanced case ascertainment among individuals with mild clinical presentations in this study. Across multiple molecular screening studies included in our meta-analysis^16,18,25,43–45^, 22% (11/49) of MPXV-positive participants who were followed up reported symptoms or received clinical diagnoses (Table S14), while a seroprevalence study from 2022 reported that only 13% of individuals with evidence of prior infection recalled recent rash or lesions^46^. In a 2022 study of US MSM, 18% and 7% of participants who experienced rash with and without fever during the outbreak peak (June-August), respectively, were tested for mpox^53^, suggesting case ascertainment may be suboptimal even among individuals who experience symptoms. Unreported infections are thus likely to include a range of clinical phenotypes spanning asymptomatic, mild, and atypical presentations with reduced likelihood of receiving diagnostic testing.

Our findings complement several observations compatible with MPXV transmission by individuals with inapparent infection. Consistent with our finding that undetected infections account for at least 31–44%, and likely 61–94%, of transmission, 67–85% of mpox cases in previous studies have reported no known exposure to individuals with mpox or mpox-like symptoms^2,12–15^. Furthermore, 28–77% of transmission events in studies of linked mpox cases involved pre-symptomatic exposures, confirming that contact with lesions is not requisite for spread^2,19,21^. Shedding of replication-competent MPXV occurs before symptom onset and during asymptomatic infection in oral, anal, and genital mucosa, providing a plausible mechanism of transmission besides skin-to-lesion contact^16–18^. Our analyses of data from external studies further confirm that MPXV c_*T*_ value distributions in anorectal specimens, and their association with successful MPXV culture, are similar in symptomatic and subclinical infections. Last, infection risk is strongly associated with condomless anal or vaginal sex^2,48,54^, which may be incompatible with the severe pain cases report while experiencing lesions^55^.

Monitoring MPXV shedding and incident disease in a single cohort, with detailed demographic and healthcare utilization data, enabled direct comparison of MPXV shedding prevalence to mpox diagnoses. Reporting multipliers based on estimates from molecular and serological surveillance studies, as well as phylogenetic analyses, validated our conclusions, and our results were robust to varied parameterizations of MPXV natural history and test specificity. However, our study has limitations. For at least three reasons, our estimates may represent a lower-bound for the extent of under-reporting of MPXV infections within the KSPC study cohort. First, studies using multiple specimen sources have identified further infections based on PCR detections from oropharyngeal specimens, urine, semen, saliva, and other sources^17,18,54^. To mitigate risk of false positive detections, we prioritized use of anorectal swabs as the most specific indicator of infection, based on reports of low c_*T*_ values, high likelihood of culturable virus, and strong associations with anti-MPXV IgG seroconversion in prior studies^16–18,25,26^. Second, available evidence suggests that greater symptom severity corresponds to longer duration of anorectal MPXV shedding^24^. Our analyses may thus underestimate the incidence of subclinical infections if underlying parameters derived from clinically-diagnosed cases overestimate the duration of asymptomatic infection. Third, within our phylogenetic analysis, sampling bias leading to over-representation of certain transmission clusters, and failure to sample others, may lead to under-estimation of sequence diversity and thus the total number of infections.

With 6 infections identified by anorectal specimen testing, our analyses aiming to distinguish vaccine effectiveness against infection and disease progression were underpowered; estimates of vaccine effectiveness could also be biased if knowledge of prior vaccination informs individuals’ and clinicians’ likelihood of diagnostic testing for mpox. Our analyses of prior molecular and serosurveillance studies relied on uncertain estimates of MSM population denominators for each setting (Table S11), likely contributing to wide variation in estimates along with other factors that may differ regionally, including testing access and risk within sexual networks. Last, caution is needed when extrapolating results of our analyses to populations at risk of clade IIb mpox besides MSM. In animal models^56,57^ and human epidemiologic studies^2,58^, sexual and non-sexual exposures are associated with distinct clinical phenotypes of mpox, and high vaccine uptake among MSM may attenuate illness severity in this population^29^.

Collectively, our results support reconsideration of control strategies in light of inapparent MPXV infections and possible cryptic circulation. Surveillance efforts relying on lesion-based clinical testing do not accurately convey individuals’ likelihood of exposure to MPXV among sexual contacts, and declining case notifications should not be interpreted as evidence of successful containment^5^. Cryptic transmission may imply that public health strategies reliant on symptom-based testing, isolation, and contact tracing offer only partial effectiveness against MPXV transmission, and the current WHO-endorsed elimination threshold^7^ may not reliably indicate cessation of spreading. After relaxation of enhanced efforts to distribute JYNNEOS during emergency phases of the mpox response, young MSM, in particular, have low vaccine coverage and may be at risk of severe mpox amid ongoing exposure to MPXV circulation. Long-term control strategies ensuring continued access to JYNNEOS and reliable monitoring of infection prevalence are needed to manage MPXV as an endemic STI among MSM.

## Methods

### Study cohort

Eligible individuals were males born between January 1, 1972, and May 1, 2008, who had been members of KPSC health plans continuously since at least 1 May, 2023 (allowing enrollment gaps <45 days), ensuring capture of prior-year healthcare utilization and recent STI history. Our age-based restrictions limited the population to individuals born after cessation of smallpox vaccination for the US general public, enabling comparison to results of other prospective testing studies^35,48^ and constraining the sample to age groups experiencing the greatest risk of mpox and other STIs^13^. We further restricted eligibility to individuals who received ≥1 anorectal *N. gonorrhoeae*/*C. trachomatis* test after 1 May, 2023, as such testing is indicated only for MSM, and who over the same period had any appointment or received any laboratory test, prescription fill, immunization, or other care at the West Los Angeles Medical Center or Los Angeles Medical Center. As these were the KPSC facilities diagnosing the greatest number of mpox cases since 2022, individuals receiving care from these facilities were expected to have the greatest likelihood of mpox diagnosis in the event of clinical illness. We report study approach and results in alignment with GATHER statement recommendations.

### Testing

Screening for STIs is recommended every 3–6 months for MSM receiving HIV pre-exposure prophylaxis and those living with HIV who continue to have new sex partners, and for MSM receiving doxycycline post-exposure prophylaxis^59,60^. Between May 29 and November 13, 2024, we obtained a total of 1190 remnant anorectal specimens submitted for *N. gonorrhoeae*/*C. trachomatis* screening among 1054 of the 7930 study cohort members during visits to the West Los Angeles or Los Angeles Medical Center facilities. Power analyses for the primary hypothesis test—that prevalence of PCR positivity in anorectal specimens was equal to the level expected when accounting for clinically-detected cases alone (0.035%)—revealed our sample size yielded >80% power at two-sided *p* < 0.05 to reject the null hypothesis under scenarios where the true ratio of infections to reported cases was >10.3 (Fig. S9). Statistical power was expected to exceed 90% if this ratio exceeded 12.8 (Fig. S9).

Samples were maintained at –20 °C and tested at the Southern California Permanente Medical Group, Regional Reference Laboratories, Chino Hills, using a clinically-validated multiplex real-time PCR that detects MPXV (clades I/II) and NVAR, as previously described^27,61^. We defined confirmed MPXV infections as those with positive results for both MPXV (c_*T*_ ≤ 36.9) and NVAR (c_*T*_ ≤ 37.6), consistent with previously-established lower limits of detection; no specimens within the study sample had inconsistent results across probes. We list primer and probe sequences for both tests in Table S14. We included an internal PCR control, i.e., a non-target nucleic acid sequence, in each reaction, and tested specimens in batches of up to 94, including positive and negative external controls within each batch.

### Sample weighting and prevalence estimation

To address differences in characteristics of individuals from whom we received or did not receive a specimen for MPXV testing during the study period, we adjusted estimates of mpox incidence and MPXV infection prevalence within the KPSC study cohort via stabilized inverse propensity weighting^62^. We computed stabilized inverse propensity-of-testing weights (${\omega }_{i}$) using a logistic regression model defining receipt of testing as the outcome; covariates included individuals’ age group, race/ethnicity, health insurance payment source, neighborhood deprivation index (a community-level proxy for individual socioeconomic status), prior-year healthcare utilization across outpatient settings and history of emergency department presentation or inpatient admission, prior-year STI testing, receipt of HIV pre-exposure prophylaxis, receipt of doxycycline post-exposure prophylaxis, receipt of JYNNEOS, prior diagnoses of HIV, syphilis, gonorrhea, chlamydia, mpox, or other STIs, and prior diagnosis of alcohol or drug abuse. We conducted all data analyses and modeling using R software (version 4.5.2; R Core Development Team).

### Time-to-event analysis for under-reporting

To generate expected infection prevalence estimates based on diagnosed mpox cases, we sampled dates of MPXV exposure (${\tau }_{E}$), shedding onset (${\tau }_{S}$), symptoms onset (${\tau }_{Y}$), and shedding cessation (${\tau }_{C}$) for cases based on their dates of positive test (${\tau }_{L}$). We estimated daily infection prevalence within the study cohort ($\pi (t)$) by dividing the sum of individuals expected to shed MPXV in anorectal specimens by the total enrolled population, applying weights as defined above, via

1 $$\pi \left(t\right)=\frac{{\sum }_{i}{\omega }_{i}{\mathbb{I}}\left[{\tau }_{S,i}\le t\right]{\mathbb{I}}\left[{\tau }_{C,i} > t\right]}{{\sum }_{i}{\omega }_{i}}.$$

We abstracted data on duration of several stages within the natural history of MPXV infection to parameterize distributions for sampling event times ${\tau }_{S}$ and ${\tau }_{C}$ from observations ${\tau }_{L}$ (Figure S2). From a study of reported cases followed prospectively from the date of diagnostic testing with repeated sampling^22^, we estimated time from symptoms onset to cessation of anorectal viral DNA detection detectable by PCR (${\tau }_{C}-{\tau }_{Y}$). From a case series of US mpox patients^1^, we estimated times from symptom onset to testing (${\tau }_{L}-{\tau }_{Y}$). Last, from a study that prospectively followed high-risk contacts of confirmed mpox cases^17^, we estimated times from exposure to onset of anorectal viral DNA detection detectable by PCR (${\tau }_{S}-{\tau }_{E}$), as well as times from exposure to symptoms onset (${\tau }_{Y}-{\tau }_{E}$). We assumed Gamma-distributed times to event and used maximum likelihood estimation to fit distribution parameters to reported data; where authors presented information on distribution quantiles only, without accompanying individual-level data, we fit Gamma distribution parameters minimizing summed squared errors between expected and reported distribution quantiles.

Our primary analysis related daily weighted, observed prevalence of infection within the anorectal swabbing study to expected infections based on reported cases in a likelihood-based framework. We defined the probability of experiencing onset of anorectal shedding on day *k* as

2 $${\lambda }_{k}=\beta \frac{{\sum }_{i}{\omega }_{i}{\mathbb{I}}\left[{\tau }_{S,i}\ge k\right]{\mathbb{I}}\left[{\tau }_{S,i} < \left(k+1\right)\right]}{{\sum }_{i}{\omega }_{i}},$$

where $\beta$ represented a multiplier relating the true number of individuals experiencing onset of shedding on day *k* to the expected number of individuals experiencing onset of shedding on day *k* based on diagnosed cases alone. Conditioned on experiencing shedding onset on day *k* < *t*, we defined the probability an individual *j* would be observed to shed on day *t* as $\Pr \left[{Z}_{j}\left(t\right)=1{|k}\right]=\Pr \left[\left({\tau }_{C}-{\tau }_{S}\right) > k\right]$. The probability of not shedding on day *t* was thus the probability of not initiating shedding by day *t*, or experiencing shedding onset and cessation before day *t*:

3a $$\Pr \left[{Z}_{j}\left(t\right)=0\right]={\prod}_{k\le t}\left(\left(1-{\lambda }_{k}\right)+{\lambda }_{k}\Pr \left[\left({\tau }_{C}-{\tau }_{S}\right) < (t-k)\right]\right),$$

3b $$\Pr \left[{Z}_{j}\left(t\right)=1\right]=1-\Pr \left[{Z}_{j}\left(t\right)=0\right].$$

The weighted likelihood all ${Z}_{j}\left(t\right)$ observations was thus

4a $$\Pr \left[{Z}_{j}\left(t\right)=1\right]=1-\Pr \left[{Z}_{j}\left(t\right)=0\right].$$

4b $${\prod}_{j}{\left({\prod}_{t}{Z}_{j}\left(t\right)\Pr \left[{Z}_{j}\left(t\right)=1\right]+\left(1-{Z}_{j}\left(t\right)\right)\Pr \left[{Z}_{j}\left(t\right)=0\right]\right)}^{{\omega }_{j}}.$$

Extending this framework for analyses comparing under-reporting between participant strata distinguished by a binary characteristic $X\in \{{{\mathrm{0,1}}}\}$, as presented in Fig. 1, we defined

5 $${\lambda }_{k,j}=\exp \left({\beta }_{0}+\left(\right.1-{x}_{j}(1-{\beta }_{1})\right)\frac{{\sum }_{i}{\omega }_{i}{\mathbb{I}}\left[{\tau }_{S,i}\ge k\right]{\mathbb{I}}\left[{\tau }_{S,i} < \left(k+1\right)\right]{\mathbb{I}}\left[{x}_{i}={x}_{j}\right]}{{\sum }_{i}{\omega }_{i}{\mathbb{I}}\left[{x}_{i}={x}_{j}\right]}.$$

We estimated the reporting multiplier $\beta$ (or ${\beta }_{0}$ and ${\beta }_{1}$, for stratified analyses) by maximum likelihood, obtaining the covariance via the inverse of the Hessian matrix.

### Alternative parameterizations of the duration of shedding

We identified two additional studies providing an indirect basis for quantifying the duration of anorectal MPXV shedding following symptoms onset. The first^23^ presented viral loads from 162 anorectal specimens collected after symptoms onset (median: 12 days; interquartile range: 6–19). We fit a regression model relating log-transformed viral load measurements to days from symptoms onset (weighting individuals by HIV status to resemble HIV status among those testing positive for MPXV in the KPSC study cohort). We confirmed via comparison of Bayesian information criterion values that analyses defining time as a continuous variable outperformed analyses applying log, quadratic, and cubic transformations of time. We then generated 10,000 draws from the prediction interval of individuals’ viral load measurements on days 1–100 after symptoms onset to estimate the probability that values would be above the lower limit of detection each day (corresponding to c_*T*_ = 37 in the primary study). We used these probabilities to define the cumulative distribution function for time to cessation of shedding, and computed first differences in the cumulative distribution function to obtain a corresponding probability mass function for days to cessation of shedding. The resulting estimate (median: 27 days; interquartile range: 17–56 days) exceeded the estimate used in primary analyses (median: 16 days; interquartile range: 12–21 days; Fig. S2), yielding a reporting multiplier of 1 in 21 (11–43) infections (Table S5).

The second study^24^ presented results from PCR testing for anorectal MPXV shedding in 18 men diagnosed with mpox, among whom all received an initial test within 1–7 days after symptoms onset, and 9 were retested within 21–32 days after symptoms onset. We fit a gamma distribution for time to cessation of shedding maximizing the likelihood of the observed numbers of individuals continuing to test positive by the time each sample was collected (median: 9.7 days; interquartile range: 6.7–14 days), yielding a reporting multiplier of 1 in 52 (25–106) infections (Table S5).

### Comparison of observed to expected infection prevalence

We conducted an alternative analysis comparing observed to expected prevalence throughout the study period. We sampled values of the mean expected prevalence throughout the study period, $\bar{\pi }={\sum }_{t}\pi (t)/\left(\max \left(t\right)-\min (t)\right)$, via draws of ${\tau }_{S}$ and ${\tau }_{C}$ for each case, and sampled values of the observed prevalence within the anorectal specimen study as $p\sim {{{\rm{Beta}}}}({\sum }_{{{{\rm{j}}}}}{\sum }_{t}{\omega }_{j}{z}_{j}(t),{\sum }_{j}{\sum }_{t}{\omega }_{j}(1-{z}_{j}(t)))$. We defined our alternative estimate for the reporting multiplier $\beta=p/\bar{\pi }$, and sampled from $\beta$ via 100,000 independent draws from *p* and $\bar{\pi }$.

### Test specificity considerations

We conducted several analyses aiming to explore potential implications of the specificity of our outcome definition (PCR-positive MPXV detection in anorectal specimens from individuals without clinically-recognized illness) for estimates of the reporting multiplier. First, we considered the relationship between PCR positivity and either culturable virus shedding or anti-MPXV IgG seroconversion, considering these outcomes to provide confirmation of MPXV infection among individuals testing positive by PCR.

For these analyses, we extracted individual-level data from all studies reporting results of PCR testing of anorectal specimens among MSM who were not clinically suspected to be experiencing mpox disease (see “Literature search”, below), including c_*T*_ values from positive anorectal specimens, results of viral culture (success or failure to isolate MPXV) from the same specimens, and results of serological testing for anti-MPXV IgG (Table S6). We defined seroconversion as anti-MPXV IgG antibody reactivity ≥21 days after a positive PCR result in individuals without prior mpox or orthopoxvirus vaccination, or a ≥4-fold rise in anti-MPXV IgG antibody titers ≥21 days after a positive PCR result for one study that did not restrict data to individuals without prior mpox or orthopoxvirus vaccination but included pre-infection sera for paired assessment^17^.

We extended our estimation framework to account for diagnosed cases’ likelihood of culturable virus shedding by defining ${\tau }_{S,i}^{V}$ as the sampled time of onset of infectious (culture-positive) virus shedding for individual *i*, such that

6 $${\lambda }_{k}=\beta \frac{{\sum }_{i}{\omega }_{i}{\mathbb{I}}\left[{\tau }_{S,i}^{V}\ge k\right]{\mathbb{I}}\left[{\tau }_{S,i}^{V} < (k+1)\right]}{{\sum }_{i}{\omega }_{i}}.$$

Whereas primary analyses considered individuals’ PCR-positive or negative status (${Z}_{j}$) as the outcome measure, we extended this framework to weight individuals’ observations by the probability of culturable virus shedding (${V}_{j}$) at observed c_*T*_ values:

7a $$\Pr \left[{V}_{j}\left(t\right)=0\right]={\prod}_{k\le t}\left(\left(1-{\lambda }_{k}\right)+{\lambda }_{k}\Pr \left[\left({\tau }_{C}-{\tau }_{S}^{V}\right) < (t-k)\right]\right)$$

7b $$\Pr \left[{V}_{j}\left(t\right)=1\right]=1-\Pr \left[{V}_{j}\left(t\right)=0\right].$$

modifying the weighted likelihood across all observations as

8 $${\prod}_{j}{\left({\prod}_{t}\Pr \left[{V}_{j}\left(t\right)=1|{{{{\rm{c}}}}}_{T,j}\right]+\left(1-{Z}_{j}\left(t\right)\right)\Pr \left[{V}_{j}\left(t\right)=0|{{{{\rm{c}}}}}_{T,j}\right]\right)}^{{\omega }_{j}}.$$

Here, we defined

9 $$\Pr \left[{V}_{j}\left(t\right)=1|{{{{\rm{c}}}}}_{T,j}\,\right]=\left\{\begin{array}{cc}{\left(1+\exp \left[-\left({\xi }_{0}+{\xi }_{1}\log \left({{{{\rm{c}}}}}_{T,j}\right)\right)\right]\right)}^{-1} & {{{\rm{if}}}} \, {{{{\rm{c}}}}}_{T,j} < 37\\ 0 & {{{\rm{if}}}} \, {{{{\rm{c}}}}}_{T,j}\ge 37,\end{array}\right.$$

with the logistic function ${(1+\exp [-({\xi }_{0}+{\xi }_{1}\log ({{{{\rm{c}}}}}_{T,j}))])}^{-1}$ yielding the probability of successful viral culture given an individual’s observed c_*T*_ value in a PCR-positive anorectal specimen. We estimated the slope (${\xi }_{1}$) and intercept (${\xi }_{0}$) parameters by fitting models via maximum likelihood to data on anorectal MPXV c_*T*_ values and viral culture results from the 11 individuals with subclinical infection identified by our literature review (Table S6), as well as data from 38 individuals with diagnosed mpox, from whom individual-level data were available on c_*T*_ values in anorectal specimens together with results of attempts to culture MPXV from these specimens (aggregated across studies in a previous review article^26^). We used the Bayesian information criterion (BIC) to assess whether model fit was improved by allowing for differences in slopes, intercepts, or both parameters for individuals with subclinical MPXV detections versus diagnosed mpox. We compared fit with models considering untransformed or log-transformed c_*T*_ values in the logistic function. Models without differences in either slope or intercept parameters across populations consistently yielded superior BIC values in comparison to those allowing parameters to differ for individuals with subclinical MPXV detections or diagnosed mpox, and *p*-values for tests of differences in slope and intercept parameters across these populations were between 0.4 and 0.8 (Table S7). This outcome suggested associations of lower c_*T*_ values with increased likelihood of successful MPXV culture from anorectal specimens were similar among individuals with diagnosed or subclinical infection.

To obtain distributions of ${\tau }_{S}^{V}$ for sampling, we fit regression models relating individual-level c_*T*_ values from anorectal specimens to time from symptoms onset, using data from the review described above^26^, identifying that a model defining log-transformed c_*T*_ values as the outcome variable, and a quadratic transformation of time as the predictor, provided the best fit to data based on BIC values. We used the fitted model parameters and residuals to sample from distributions of individuals’ anticipated c_*T*_ values at time points through 50 days after symptoms onset, and estimated probabilities for culturable virus shedding at each of the sampled c_*T*_ values to recover distributions of individuals’ probability of culturable virus shedding as a function of time (Fig. S3).

For analyses adjusting estimates of subclinical infection prevalence for the proportion of PCR-positive anorectal MPXV detections associated with seroconversion, we defined $\zeta \sim {{{\rm{Beta}}}}(8,1)$ according to the data aggregated from previous studies monitoring seroconversion (Table S7). We defined the reporting fraction as $\zeta \beta$ to exclude the proportion ($1-\zeta$) of PCR-positive anorectal detections not expected to result in seroconversion.

Last, we considered the possibility of false-positive PCR detections. Previous real-world validation of the study assay^27^ observed *x* = 0 positive results among *n* = 64 true negative specimens; parameterizing a corresponding negative binomial distribution under differing specificity values (*p*) ranging from 0.1% to 15% for a single test, the maximum likelihood value for single-test specificity was 100%, and single-test specificity was expected to take values ≥ 99.9%, ≥98.8%, and ≥94.8% with likelihood of 90%, 50%, and 5%, respectively. We quantified the positive predictive value (PPV) of a dual-positive result for MPXV and NVAR as

10 $${{{\rm{PPV}}}}=\frac{{{{\rm{Prevalence}}}}}{{{{\rm{Prevalence}}}}+\left(1-{{{\rm{Prevalence}}}}\right){\left(1-{{{\rm{Specificity}}}}\right)}^{2}}$$

under scenarios with true prevalence equal to 0.91% (as estimated in the study) or 0.455% (corresponding to a scenario where half of all detections were false positives). We repeated our primary analyses, multiplying infection prevalence by the resulting dual-positive PPV to generate corrected estimates accounting for varying test specificity (Fig. S4).

We also used the distribution of quantitative c_*T*_ values for the MPXV-specific and NVAR probes to assess potential false-positive signals. Whereas c_*T*_ values were expected to be correlated if measuring true MPXV-associated nucleic acids, spurious signals leading to false positive results were expected to have no association across probes. To simulate the expected difference in MPXV-specific and NVAR c_*T*_ values under a scenario of independence (due to false positive results), we took 7 draws each from the distributions of observed c_*T*_ values for each probe. We computed mean differences in c_*T*_ between the two probes, and measured the probability of a mean difference as extreme as that observed in our sample as

11 $${p}_{{{{\rm{Two}}}}-{{{\rm{sided}}}}}=2\times \Pr \left({\sum }_{i}|{{{{\rm{c}}}}}_{T}^{{{{\rm{Simulated}}}}}{\left({{{\rm{MPXV}}}}\right)}_{i}-{{{{\rm{c}}}}}_{T}^{{{{\rm{Simulated}}}}}{\left({{{\rm{NVAR}}}}\right)}_{i}|\right.\\ \left.\le {\sum }_{i}|{{{{\rm{c}}}}}_{T}^{{{{\rm{Observed}}}}}{\left({{{\rm{MPXV}}}}\right)}_{i}-{{{{\rm{c}}}}}_{T}^{{{{\rm{Observed}}}}}{\left({{{\rm{NVAR}}}}\right)}_{i}|\right)\,$$

### Incidence rate estimation

We estimated incidence rates of MPXV infection within the reweighted sample, adjusted for reporting, as $\beta {\sum }_{i}{\omega }_{i}{y}_{i}/{\sum }_{i}{\omega }_{i}\min \left({\tau }_{Y,i},{\tau }_{F,i}\right)$, where ${\tau }_{F,i}$ was the earliest of the date of death, disenrollment, or study termination for individual *i*.

To contextualize our estimates of the incidence rate of MPXV infection, we also estimated incidence rates for infection with *N. gonorrhoeae*, *C. trachomatis*, and *T. pallidum*. We defined *N. gonorrhoeae* and *C. trachomatis* infections as any positive molecular test result separated by ≥30 days from any prior positive test result or diagnosis; we defined *T. pallidum* infection based on positive results for *Treponema*-specific IgG or IgM. To correct for potential under-ascertainment of these other STIs, we fit Poisson regression models relating individuals’ rates of infection during the study period to their screening frequency in the prior year. Models defined counts of unique diagnoses (separated by ≥30 days from a previous diagnosis) with each infection during the study period as the outcome variable and included log-transformed person-time at risk as an offset term. The primary exposure of interest was the prior-year frequency of testing (between May 1, 2023, and April 30, 2024). For *N. gonorrhoeae* and *C. trachomatis*, *w*e defined receipt of ≥4 tests as the “best-case” referent category based on guidelines that MSM should be screened up to every 3 months for continuation of HIV PrEP and doxycycline post-exposure prophylaxis^59,60^; for *T. pallidum*, we defined receipt of ≥2 tests as the “best-case” referent category due to the lower rate of infection and lack of evidence for increased incidence of syphilis diagnoses with testing at shorter intervals in the study population. To ensure measured testing behavior reflected engagement with screening rather than test-seeking for symptomatic illness or known exposures, models adjusted for individuals’ receipt of any gonorrhea diagnosis or chlamydia diagnosis in the prior year, as well as age group, insurance source, receipt of HIV PrEP and doxycycline post-exposure prophylaxis, receipt of JYNNEOS, HIV infection, and prior syphilis diagnosis. As *T. pallidum* infection is diagnosed by serology, we limited analyses for this infection to individuals with no prior history of syphilis diagnoses or positive test results.

Regression parameters ${\alpha }_{1}$ and ${\alpha }_{2}$ compared rates of STI diagnoses associated with prior-year receipt of 0–1 tests versus ≥4 tests and 2–3 tests versus ≥4 tests, respectively, and thus represented reporting ratios for STI compared to a scenario where individuals’ testing frequency met CDC recommendations. We defined the correction factor relating true rates of infection to rates of diagnoses as

12 $$\epsilon=\frac{{\sum }_{i}{\omega }_{i}\left({\alpha }_{1}^{-1}{\mathbb{I}}\left[{a}_{i} < 2\right]+{\alpha }_{2}^{-1}{\mathbb{I}}\left[{2\le a}_{i} < 4\right]+\Pr {\mathbb{I}}\left[{a}_{i}\ge 4\right]\right)}{{\sum }_{i}{\omega }_{i}}$$

and multiplied incidence of rates of STI diagnoses by $\epsilon$ to obtain incidence rates of infection. The corresponding equation for corrected incidence rates of *T. pallidum* infection was

13 $$\epsilon=\frac{{\sum }_{i}{\omega }_{i}\left({\alpha }_{1}^{-1}{\mathbb{I}}\left[{a}_{i} < 2\right]+{\alpha }_{2}^{-1}{\mathbb{I}}\left[{a}_{i}\ge 2\right]\right)}{{\sum }_{i}{\omega }_{i}}.$$

### Vaccine effectiveness estimation: overview

We aimed to estimate three classes of vaccine direct effects, comparing counterfactual risk of mpox-related clinical outcomes among individuals who received JYNNEOS versus those who did not receive JYNNEOS. Following Halloran et al.^63^, we considered that vaccination could protect against diagnosed mpox by: reducing individuals’ susceptibility to acquiring MPXV infection, given exposure, by a factor ${{{{\rm{VE}}}}}_{S}=\left(1-{\theta }_{S}\right)\times 100 \%$; and by reducing individuals’ risk of progression to diagnosed mpox, given MPXV infection, by a factor ${{{{\rm{VE}}}}}_{P}=\left(1-{\theta }_{P}\right)\times 100 \%$. The reduction in risk of diagnosed mpox, given vaccination, was thus ${{{{\rm{VE}}}}}_{D}=\left(1-{\theta }_{S}{\theta }_{P}\right)\times 100 \%$.

We used a case-control design to estimate ${{{{\rm{VE}}}}}_{D}$, and confirmed results of this primary analysis using a negative control-adjusted prospective cohort framework. We also estimated ${{{{\rm{VE}}}}}_{S}$ using two analysis frameworks, based on both case-control and prospective-cohort designs. We used the results of these analyses to estimate ${{{{\rm{VE}}}}}_{P}$, defining

14 $${{{{\rm{VE}}}}}_{P}=\left(1-{\theta }_{P}\right)\times 100\%= \left(1-\frac{{\theta }_{S}{\theta }_{P}}{{\theta }_{S}}\right)\times 100\%= \left(1-\frac{100-{{{{\rm{VE}}}}}_{D}}{100-{{{{\rm{VE}}}}}_{I}}\right)\times 100\%.$$

Our analyses considered pre-exposure JYNNEOS vaccination only, as no post-exposure prophylaxis with JYNNEOS occurred among cohort members during the study period. All previously-administered JYNNEOS doses were considered pre-exposure doses with respect to follow-up for mpox during the study period.

### Estimation of vaccine effectiveness against diagnosed mpox

Our primary analysis estimating ${{{{\rm{VE}}}}}_{D}$ used a case-control framework comparing adjusted odds of prior vaccination among cases diagnosed with laboratory-confirmed mpox to controls diagnosed with laboratory-confirmed gonorrhea during the study period. This strategy was anticipated to mitigate confounding based on the expectation that receipt of JYNNEOS could be associated with individuals’ risk of STI exposure as well as their engagement with sexual health services and likelihood of being diagnosed, if infected^64,65^; whereas JYNNEOS would not be expected to alter individuals’ risk of gonorrhea, gonorrhea cases were expected to resemble mpox cases in sexual risk characteristics and healthcare-seeking behavior. We defined the product ${\theta }_{S}{\theta }_{P}$ as the adjusted odds ratio of prior JYNNEOS vaccination (receipt of any doses, 1 dose, or ≥2 doses) among mpox cases relative to controls diagnosed with gonorrhea, and estimated this term via conditional logistic regression. We defined matching strata on individuals’ HIV infection status and (among HIV-negative individuals) receipt or non-receipt HIV PrEP; receipt of doxycycline post-exposure prophylaxis; and history of any syphilis diagnosis. Models further controlled for individuals’ age group, receipt of *N. gonorrhoeae*/*C. trachomatis* testing in the prior year, and commercial or non-commercial insurance source (expected to proxy socioeconomic status) via covariate adjustment.

We conducted a sensitivity analysis leveraging the prospective design of the study and estimating ${{{{\rm{VE}}}}}_{D}$ via a negative control-corrected incidence ratio, following a previously-described framework^66^ recently validated in a randomized controlled trial setting^67^. This analysis used a two-stage approach; we first estimated adjusted incidence rate ratios (IRRs) comparing incidence of mpox diagnoses and gonorrhea diagnoses among JYNNEOS recipients (any receipt, 1 dose receipt, or ≥2 dose receipt) versus non-recipients. Considering that any apparent association of JYNNEOS vaccination with gonorrhea incidence would signify bias due to differential sexual risk and healthcare-seeking behaviors among vaccine recipients and non-recipients, we used the estimated IRR for gonorrhea to adjust the causal effect estimate for the IRR of mpox associated with JYNNEOS vaccination, such that

15 $${{{{\rm{VE}}}}}_{D}=\left(1-{\theta }_{S}{\theta }_{P}\right) \times 100\%=\left(1-{{{{\rm{IRR}}}}}_{{{{\rm{mpox}}}}}/{{{{\rm{IRR}}}}}_{{{{\rm{gonorrhea}}}}}\right)\times 100\%.$$

We estimated each IRR via Poisson regression models, defining mpox or gonorrhea diagnoses as the outcome variables and including log person-time at risk as an offset, and adjusted for all risk factors included in the primary analysis models (HIV infection, receipt of HIV PrEP, receipt of doxycycline post-exposure prophylaxis, prior syphilis diagnosis, age group, prior-year gonorrhea testing, and commercial insurance source) as covariates.

### Estimation of vaccine effectiveness against MPXV infection

Our first analysis estimating ${{{{\rm{VE}}}}}_{S}$ defined ${\theta }_{S}$ as the adjust odds ratio of prior JYNNEOS receipt (any doses, one dose, or two doses) or non-receipt comparing individuals within the anorectal specimen testing study who received positive MPXV results to those receiving negative results. We considered the outcome of positive results from asymptomatic rectal specimen based on the assumption that infections captured within the testing study represented the source population from which diagnosed mpox cases would be drawn; although none of the 6 individuals receiving positive results in our study ultimately progressed to diagnosed illness, such progression has been identified in other studies employing similar designs (Table S13). We fit ${\theta }_{S}$ using conditional logistic regression models defining positive or negative test results from each specimen as the outcome, defining matching strata for individuals’ HIV infection status and (among HIV-negative individuals) receipt or non-receipt HIV PrEP, receipt of doxycycline post-exposure prophylaxis, and history of any syphilis diagnosis, consistent with our analyses estimating VE_*D*_. We included data from all specimens, and used the sandwich estimator to adjust variance estimates for repeat sampling of individuals.

We also estimated ${{{{\rm{VE}}}}}_{S}$ via the adjusted IRR of MPXV infection comparing previously-vaccinated individuals to unvaccinated individuals. These analyses applied reporting multipliers ($\beta$ parameters, as described above) specific to the vaccinated and unvaccinated populations in defining MPXV incidence rates for the comparator populations.

### Examining the role of undetected infections in transmission: overview

We undertook two analyses aiming to clarify the potential contribution of individuals with undetected MPXV infections to transmission. The first quantified the minimum degree of dispersion (maximum value of *k*) corresponding to a scenario where individuals with undetected infection make no contribution to transmission, and compared this value to previously-reported estimates of *k* for MPXV based on prior phylogenetic studies^39–41^. The second analysis estimated the proportion of infections caused by individuals with undetected infection under scenarios with $k=0.3$, consistent with these prior estimates^39–41^.

### Minimum dispersion under scenarios without transmission by individuals with undetected infection

We first explored implications of the hypothesis that individuals who receive mpox diagnoses account for all transmission by estimating values of a dispersion parameter *k* that would correspond to this scenario. We measured the dispersion parameter as

16 $$k=\frac{R+{R}^{2}}{{{{\mathrm{var}}}}\left(U\right)}.$$

where *R* specified the reproduction number, or mean number of secondary infections (*U*) resulting from each index infection^36–38^. Greater values of *k* correspond to lower degrees of dispersion in the number of infections caused by each index case; *k* approaches 0 as variance in *U* approaches $\infty$, and *k* approaches $\infty$ as variance in *U* approaches 0. Maximum values of *k*, corresponding to the minimum degree of dispersion, arise under a scenario assuming uniform numbers of secondary infections caused by individuals with detected infections:

17 $$U=\left\{\begin{array}{cc}R\beta & {{{\rm{with}}}\; {{\rm{probability}}}}\, 1/\beta \\ 0 & {{{\rm{with}}}\; {{\rm{probability}}}}\,1-1/\beta .\end{array}\right.$$

We sampled 10,000 distributions of *U* under each for $R\in 0.5,\,0.6,\,\ldots 3.5$, and repeated analyses under a scenario where only half of individuals at risk for transmitting infection received diagnoses (e.g., due to lack of clinical recognition of symptoms), where

18 $$U=\left\{\begin{array}{cc}R\beta /2 & {{{\rm{with}}}\; {{\rm{probability}}}}\,2/\beta \\ 0 & {{{\rm{with}}}\; {{\rm{probability}}}}\,1-2/\beta .\end{array}\right.$$

Across the range of *R* values considered, our analyses yielded maximum *k* estimates that remained consistently below the range of previously estimates^39–41^, suggesting that the hypothesis of transmission only by diagnosed mpox cases implies an implausible degree of variation in individuals’ risk of transmitting MXPV.

### Contribution of undetected infections to transmission with realistic dispersion

We next sought to estimate the proportion of transmission that could be attributed to individuals with undetected infections under scenarios with *k* = 0.3, consistent with published estimates^39–41^. Defining $U \sim {{{\rm{Negative\; Binomial}}}}(R\in 0.7,\,0.8,\,\ldots,\,3.5,{k}=0.3)$, we drew values *U* and considered differing schemes for linking secondary infections to diagnosed or undiagnosed index cases.

Our first approach maximized the number of secondary infections attributed to diagnosed index cases. Defining $D=N/\beta$ as the number of diagnosed cases among *N* infections, for a sorted vector ${U}^{{\prime} }$ (with ${u}_{1}^{{\prime} }\ge {u}_{2}^{{\prime} }\ge \cdots \ge {u}_{N}^{{\prime} }$), the proportion of infections attributable to diagnosed cases was ${\sum }_{i=1}^{D}{u}_{i}^{{\prime} }/{\sum }_{i}^{N}{u}_{i}$, and the proportion attributable to undetected infections was $1-({\sum }_{i=1}^{D}{u}_{i}^{{\prime} }/{\sum }_{i}^{N}{u}_{i})$.

To relax this deterministic sorting framework, we next considered scenarios where individuals’ likelihood of transmission was associated with being a diagnosed case. Defining $Y$ as a vector indicating receipt of a diagnosis or no receipt of a diagnosis for a simulated population of cases (with probability $1/\beta$ of receiving a diagnosis), we defined ${\mathbb{E}}\left[U\right]=\exp \left({\delta }_{0}+{\delta }_{1}Y\right)$ for ${\delta }_{1}$ values corresponding to 2-, 4-, 10-, and 20-fold greater numbers of secondary infections attributable to individuals diagnosed with mpox compared to undetected MPXV infections. We defined ${\delta }_{0}={{{\mathrm{ln}}}}\left[R/\exp \left({\delta }_{1}/\beta \right)\right]$ and solved via gradient descent for values of $\theta$ satisfying the condition

19a $${u}_{i} \sim {{{\rm{Negative\; Binomial}}}}\left(\exp \left[{\delta }_{0}+{\delta }_{1}{y}_{i}\right],\theta \right)$$

19b $$\frac{R+{R}^{2}}{{{{\mathrm{var}}}}\left(U\right)}=0.3$$

for sampled $Y$ vectors of length 10,000.

Drawing from the resulting joint distributions of *Y* and *U*, the proportion of transmission attributable to individuals with diagnosed mpox was ${\sum }_{i}{y}_{i}{u}_{i}/{\sum }_{i}{u}_{i}$, while the proportion attributable to individuals with undetected infections was ${\sum }_{i}\left(1-{y}_{i}\right){u}_{i}/{\sum }_{i}{u}_{i}$.

### Elimination threshold assessment

Current WHO guidance defines mpox elimination in settings with surveillance capacity for confirmation of suspect cases, without zoonotic reservoirs for MPXV, and where outbreaks are concentrated in sexual networks, as ≥3 months without reported mpox cases^7^. However, from a binomial distribution parameterized using our estimated reporting multiplier (1 in 33 [16–68]), the probability of observing *x* = 0 diagnosed cases would be ≥50% with *n* equal to as many as 23 (11–47) true infections, and ≥5% with *n* as great as 98 (48–202) true infections. We used the modeling framework described above to estimate the probability that an outbreak was truly controlled under a scenario where ≥3 months had passed without additional notified cases.

We simulated transmission trees using a negative-binomial offspring distribution for values of *R* between 0.7 and 3.5 and *k* = 0.3, using prior meta-analytic estimates of the serial interval distribution for clade IIb mpox in outbreaks driven by sexual transmission to draw inter-event times^68^. We generated 100,000 simulated transmission chains for each value of *R*, and sampled cases receiving diagnoses among all simulated infections using our primary reporting multiplier estimate, halting simulations after either 2 years or the occurrence of >20,000 infections. We tabulated instances where 90 days passed after a notified case, with no other case notifications over this interval, and evaluated whether transmission chains had in fact concluded by assessing whether transmission chains included additional infections >90 days after the date of the last notified case. We stratified probabilities that transmission had concluded according to *R* and the total number of notified cases before onset of the monitoring period.

### Validation of under-reporting via meta-analysis of data from other settings

We conducted a systematic literature review of prior studies employing prospective testing for MPXV infection in anorectal specimens or anti-MPXV antibodies via blood specimens. We compared observed prevalence within the study populations to expected prevalence in the corresponding geographic areas based on local mpox case notification data from each setting, using a framework resembling our primary analyses within the KPSC study cohort. Analyses included corrections for potential differences in MPXV infection risk among individuals recruited in testing studies versus the general MSM population.

### Literature search

We searched PubMed for the terms (mpox OR monkeypox) AND (prevalen* OR inciden* OR surveill*) in full-length research articles published in English between 1 April, 2022 and 31 May, 2025, yielding 580 articles. We reviewed articles to identify those presenting results of studies that prospectively or retrospectively evaluated (*a*) prevalence of MPXV detection via anorectal samples among MSM who were not clinically suspected to be experiencing mpox disease, or (*b*) prevalence of anti-MPXV IgG detection among MSM who were not clinically suspected to be experiencing mpox disease, and who were not recruited based on clinical knowledge or suspicion of prior MPXV infection. We limited studies to those undertaken in regions where mpox was not understood to be endemic prior to 2022. Studies that did not exclude non-MSM from participation, or that did not present data stratified by MSM status, were eligible for inclusion if ≥70% of the sample were cisgender MSM or reported transgender/non-binary gender expression. Due to the rare nature of the outcome, we excluded studies that enrolled <100 individuals. To supplement our search, we conducted forward and backward citation tracking in Google Scholar from articles that met inclusion criteria to identify additional articles for screening.

To ensure comparability with our findings, we included results from anorectal specimens only when studies presented results from both anorectal and oropharyngeal specimen testing. We excluded studies that presented results from other specimen types (e.g., urine or saliva) without disaggregation of positive and negative results by specimen source. For serological studies that included vaccinated persons, we restricted eligibility to those using assays distinguishing naturally-acquired from MVA-induced antibody responses.

### Comparison of observed-to-expected prevalence in other settings

Within each setting where infection prevalence or seroprevalence studies were undertaken, we obtained publicly-reported mpox case counts at the finest-available temporal and spatial resolution. We estimated expected prevalence of infection, based on observed cases alone, by sampling dates of exposure (${\tau }_{E}$), onset of MPXV shedding (${\tau }_{S}$), and cessation of shedding (${\tau }_{C}$), as described above for analyses within the KSPC study cohort; for settings where cases were aggregated by dates of reporting rather than dates of testing, we assumed a 1-day lag from the date a diagnostic test was performed to the reporting date. Where location-specific data were available on the proportion of mpox cases occurring among MSM, we multiplied daily case counts by this proportion to account for cases among MSM only; where such data were not available, we assumed MSM accounted for 95% of cases^1^. For settings aggregating case notifications by week, we modeled daily diagnoses over the corresponding weeks as 1/7th of the weekly total.

We estimated expected daily infection prevalence, accounting for reported cases alone, by dividing the total number of individuals expected to shed MPXV each day by estimates of the corresponding MSM population size. We obtained MSM population denominators for each geographic area via a literature search, using census data from each region to project changes in population size from historical estimates (Table S11). For serological studies, we assumed onset of detectable IgG at time of shedding cessation, based on previous evidence of times to seroresponse after natural infection^69,70^ and vaccination^71^. We propagated uncertainty across 100,000 sampled time series of the daily prevalence of infection and seropositivity, and defined expected period-wide prevalence or seroprevalence as the mean of daily values over the enrollment period of each study, as described above for analyses comparing observed to expected prevalence within the KPSC study cohort.

### Risk normalization for reporting multipliers in other settings

Consistent with the rationale for inverse propensity weighting in the KPSC study, we expected that comparisons of MPXV infection prevalence or seroprevalence within study populations to mpox incidence among MSM in the surrounding geographic area could be biased by differences in risk among MSM enrolled in clinic-based samples and general-population samples. We used a two-stage procedure to correct for such bias. Because prior studies have provided estimates of the association of mpox risk with MSM’s number of anal sex partnerships over the preceding 21 days^2,72^, and because total numbers of anal sex partnerships over specified intervals are a frequently-collected and frequently-reported characteristic of participants in sexual health research, we sought to reconstruct distributions of (*a*) anal sex partnerships per 3-week interval among MSM in clinic-based and general-population samples, and (*b*) relative risks of mpox, based on these sampled anal sex partnership counts.

We identified four studies reporting on anal sex partnership counts among MSM recruited in sexual health facilities^73–75^; we constrained our search for studies to those undertaken within the US or Western Europe, as all included studies of MPXV prevalence were undertaken in these settings. We considered the sample enrolled in the ARTnet study as a reference population^76,77^ between July 2017 and January 2019. The sample for this study was recruited online among MSM directly after participation in the American Men’s Internet Study^78^, an annual online behavioral survey recruiting MSM via email blasts and banner advertisements on websites or mobile phone applications targeting differing audiences (gay social networking, gay general interest, general social networking, and geospatial social networking). While the lack of a reference frame precludes comparison of the characteristics of the surveyed population to all MSM, consistency of STIs incidence within this study sample and US surveillance data suggests risk characteristics of the population are representative of those of MSM in general. Specifically, HIV prevalence within the study sample was 10.8%, comparable to the estimate of 11.1% prevalence of diagnosed HIV infection among all US MSM^79^. Gonorrhea incidence within the sample was 5.07 cases/100 person-years, comparable to the estimate of 5.17 cases/100 person-years among MSM^80^. Additionally, prior comparative studies have reported that sexual risk behaviors are similar in MSM samples recruited through such online surveys and those recruited via physical venue-based sampling^81^.

To account for the right-tailed shape of the distribution of individuals’ reported anal sex partnerships, we considered this count to follow a negative binomial distribution for the recall interval ($\tau$ days) used in each study, ${Z}_{\tau } \sim {{{\rm{Negative\; Binomial}}}}(p,r)$. We parameterized negative binomial distributions using either reported means and variances of distributions or via least-squares fitting to reported distribution quantiles. We sampled from the fitted distributions for pseudo populations of 1 million individuals and projected total partnership counts for individuals over a 3-week interval by multiplying the resulting samples by the factor $21/\tau$. To accommodate non-integer valued results, we drew counts probabilistically based on the remainder, with ${z}_{21}\left(i\right)={{{\rm{Floor}}}}\left[{z}_{21}^{*}(i)\right]+{{{\rm{Bern}}}}\left({z}_{21}^{*}\left(i\right)-{{{\rm{Floor}}}}\left[{z}_{21}^{*}(i)\right]\right)$. We used the association of anal sex partnership counts over a 21-day period from a test-negative design case-control study of mpox as the primary basis for assigning risk based on partnership counts^2^. Compared to individuals who reported 0–1 anal sex partners in the 21 days preceding symptoms, adjusted odds ratios associated with reporting 2–3 partners and ≥4 partners were 2.2 (1.0–4.8) and 3.8 (1.7–8.8). We used these estimates to define and sample from probability distributions for individuals’ relative risk of mpox $\pi (i)$ given their sampled partnership history, $f(\pi (i)|{z}_{21}\left(i\right))$, considering the adjusted odds ratio and relative risk to be equivalent under the test-negative design framework^82^. We repeated this analysis using alternative, model-based estimates of the relative risk of mpox given partnership history^72^, which yielded similar risk multipliers for adjustment.

### Pooling of reporting multipliers

We pooled estimates of the (log-transformed) reporting multiplier across all validation samples (molecular surveillance studies; serosurveillance studies) using inverse variance-weighted random effects models^83^, for both unadjusted and risk-adjusted analyses (Table S11).

### Validation of under-reporting via phylogenetic analyses: overview

Last, we sought to estimate reporting multipliers within Los Angeles County by comparing the number of diagnosed cases to an estimate of the total MPXV viral population size generated by phylogenetic analyses. As detailed below, our workflow comprised generating a maximum-likelihood phylogeny of MPXV, generating estimates of the sampling proportion ($\rho$) accounting for both local transmission and repeated introductions of MPXV into Los Angeles County, and deriving the reporting multiplier $\beta$ from our estimates of *p*. Here, *p* is defined as the ratio of the number of sequenced mpox cases to the effective population size (${{N}_{{{{\rm{sequenced}}}}}/N}_{{{{\rm{eff}}}}}$) of MPXV in Los Angeles County^51,84,85^. We describe these steps below together with a simulation study validating the framework for estimating $\rho$.

### Phylogenetic inference

We used all available MPXV clade IIb genomes available from Genbank on January 20, 2025, and identified local transmission clusters in Los Angeles County under a framework outlined in a previous study of MXPV phylodynamics^40^. We created a temporally-resolved phylogeny using a modified version of the Nextstrain^86^ mpox workflow (https://github.com/nextstrain/mpox), which aligns sequences against the MK783032 (collection date: Nov. 2017) reference using nextalign^87^. Briefly, this workflow included inferring a maximum-likelihood phylogeny using IQ-TREE^88^ with a general time-reversible nucleotide substitution model, estimating molecular clock branch lengths via TreeTime^89^. The resulting phylogeny is available from https://nextstrain.org/groups/blab/mpox/allcladeIIseqs.

Our analyses included 497 de-duplicated MPXV sequences for Los Angeles County cases. Of this total, 271 were sampled between May 21, 2022 (the date of the first notified mpox case in California associated with the ongoing clade IIb outbreak) and October 14, 2022, and 226 were sampled between October 15, 2022, and December 31, 2024. The Los Angeles County Department of Public Health (LACDPH) sequences mpox cases diagnosed by healthcare providers in Los Angeles County, we assume that any genome sequenced by LACDPH was sampled locally. As the California Department of Health (CDPH) supports sequencing efforts only for local public health departments without internal sequencing capacity, we assumed additional MPXV sequences (*N* = 222) sequenced by CDPH were sampled in locations within California outside of Los Angeles County. We clustered all Los Angeles County sequences based on inferred internal node location using a parsimony-based approach to reconstruct the locations of internal nodes^40,52^, identifying 287 unique clusters among the observed sequences for which a shared common ancestor was most likely to have been in Los Angeles County.

We inferred the time-varying effective reproduction number (${R}_{t}$), $\rho$, and phylogenies from all local clusters jointly using a birth-death skyline model^49^, assuming that local outbreak clusters represented independent stochastic realizations of a transmission process with the same parameters^90^. We assumed ${R}_{t}$ to be piecewise-constant in intervals of 7 days, with

20 $$\log \left(\frac{{R}_{t+7}}{{R}_{t}}\right) \sim {{{\rm{Normal}}}}\left(0,{\sigma }^{2}\right).$$

We estimated *σ* via Markov chain Monte Carlo sampling (MCMC) using BEAST2 (version 2.7.8)^50^. We allowed an 8-day mean infectious period (${T}_{g}$), reflecting our assumption that detectable shedding of MPXV genetic material outlasts individuals’ period at risk of onward transmission (as described previously^22^ and accounted for in our estimates of expected infection prevalence in anorectal specimens; Fig. S2). We employed a strict molecular clock with a fixed value of 0.00006^39^, and a HKY + Γ nucleotide substitution model with a fixed $\kappa=18.5$^91^. Of note, the birth-death skyline model conditions on survival^49^, meaning that it computes the probability of observing a phylogenetic tree conditional on observing at least one lineage, and assumes the host population to be unstructured. Code for implementing this adapted version is available from https://github.com/nicfel/bdsky.

### Validation of estimation framework

Birth-death skyline models assume unstructured populations, and hidden population structure may bias inference results. Further, they do not model skewed offspring distributions, as is the case for mpox transmission. To test the validity of our approach to estimating $\rho$ under realistic mpox transmission dynamics, we simulated phylogenetic trees under using a stochastic, individual-based Susceptible-Exposed-Infectious-Recovered (SEIR) model with superspreading^92^. We modeled importation assuming a constant force of introduction per unit time, and (as described above) assumed an offspring distribution with $U \sim {{{\rm{Negative\; Binomial}}}}(R,{k})$. Primary analyses defined $k=0.3$, consistent with previous estimates for MPXV^39–41^; we additionally explored performance of the method with $k=0.1$ (represent extreme dispersion) and $k=1$ (representing low dispersion). To approximate real-life sampling dynamics, we parameterized time to sampling via the estimated time to healthcare presentation among UK mpox patients in 2022^93^. We also tested performance under lower sampling schemes, reducing the sampling by 50% and 90%, relative to observed levels. We recorded structured phylogenetic trees from model output and simulated corresponding genetic sequences using Seq-Gen (version 1.3.4)^94^, assuming an HKY substitution model, a genome size of 197,000 bps, and a clock rate of 0.00006. We then ran simulated outputs through our multi-tree birth-death modeling pipeline and compared the estimated sampling proportion to input parameter values (Fig. S7).

### Relating the sampling proportion to the number of infections

We used a previous derivation^51^ of the relationship between ${N}_{{{{\rm{eff}}}}}$ and the total number of infections, ${N}_{{{{\rm{tot}}}}}$, to generate reporting fractions based on our estimates of $\rho$ from each analysis period (May 21 through October 14, 2022, and October 15, 2022, through December 31, 2024). Defining a coalescence rate $\lambda=1/\left({N}_{{{{\rm{eff}}}}}{T}_{g}\right)$ and an offspring distribution $U \sim {{{\rm{Negative\; Binomial}}}}(R,k)$,

21 $$\lambda=\frac{R(1+1/k)}{{N}_{{{{\rm{tot}}}}}{T}_{g}},$$

such that ${N}_{{{{\rm{tot}}}}}=R\left(1+1/k\right){N}_{{{{\rm{eff}}}}}$. With $\rho={N}_{{{{\rm{sequenced}}}}}/{N}_{{{{\rm{eff}}}}}$, we obtain numerical estimates of ${N}_{{{{\rm{tot}}}}}$ during each analysis period as

22 $${N}_{{{{\rm{tot}}}}}=\frac{R\left(1+\frac{1}{k}\right){N}_{{{{\rm{sequenced}}}}}}{\rho }$$

and obtain conditional values of ${\beta }_{R}$ by dividing total diagnosed cases by ${N}_{{{{\rm{tot}}}}}$. We present estimates of ${\beta }_{R}$ across a range of $R$ estimates. Additionally, we obtain period-specific pooled estimates for $\beta$, weighting these conditional values by $\Pr ({R}_{t}=R)$ for mean ${R}_{t}$ values over each analysis period, i.e.

23 $${\beta }_{{{{\rm{pooled}}}}}={\sum }_{i}{\beta }_{{R}_{i}}\Pr ({R}_{t}={R}_{i}).$$

### Ethics

The study was reviewed and approved by the Kaiser Permanente Southern California Institutional Review Board, with a waiver of the requirement for informed consent due to secondary use of electronic health records and clinical specimens. The study was conducted in compliance with all relevant ethical regulations.

### Reporting summary

Further information on research design is available in the Nature Portfolio Reporting Summary linked to this article.

## ^Supplementary information^

Supplementary Information

Reporting Summary

Transparent Peer Review file

## Source data

Source Data

## Data Availability

Individual-level testing and clinical outcomes data reported in this study (Figs. 1–2) are not publicly shared due to privacy protections for patient electronic health records. Individuals wishing to access disaggregated data, including data reported in this study, should submit requests for access to sara.y.tartof@kp.org. Requests will receive a response within 14 days. De-identified data (including, as applicable, participant data and relevant data dictionaries) will be shared upon approval of analysis proposals with signed data-access agreements in place. Source data not requiring information from patient electronic health records are provided for analyses presented in Figs. 3–5. Source data are provided with this paper.

## Code availability

Analysis code is publicly available without restriction from the corresponding author’s “mpoxTesting” GitHub repository (joelewnard/mpoxTesting). The version of record is available from:10.5281/zenodo.19488428^95^.
