# Supplementary material for: Extensive cryptic circulation sustains mpox among men who have sex with men
Source: Nat Commun. 2026 May 13;17:4198. doi: 10.1038/s41467-026-72749-2 (PMC13172365; doi:10.1038/s41467-026-72749-2)
Supplement: Supplementary file 1 — Supplementary Information [file 41467_2026_72749_MOESM1_ESM.pdf]

Supporting information: Extensive cryptic circulation sustains mpox among men who have sex with men

Joseph A. Lewnard[1,2], Miguel I. Paredes[3], Matan Yechezkel[1], Gregg S. Davis[4], Vennis Hong[4], Jessica Skela[4], Utsav Pandey[5,6], Noah T. Parker[1,7], Lauren C. Granskog[1], Magdalena E. Pomichowski[4], Iris Anne C. Reyes[4], Isabel Rodriguez-Barraquer[8,9], Nicola F. Müller[8], Sara Y. Tartof[4,10]

1. Division of Epidemiology, School of Public Health, University of California, Berkeley; Berkeley, California 94720, United States
2. Center for Computational Biology, College of Computing, Data Science, and Society, University of California, Berkeley; Berkeley, California 94720, United States
3. Vaccine and Infectious Disease Division, Fred Hutchinson Cancer Center; Seattle, Washington 98109, United States
4. Department of Research & Evaluation, Kaiser Permanente Southern California; Pasadena, California 91101, United States
5. Regional Reference Laboratories, Kaiser Permanente Southern California; Chino Hills, California 91709, United States
6. Department of Clinical Science, Bernard J. Tyson School of Medicine; Pasadena, California 91101, United States
7. Department of Epidemiology, Johns Hopkins Bloomberg School of Public Health, Baltimore, Maryland 21205, United States
8. Division of HIV, Infectious Diseases, and Global Medicine, Department of Medicine, University of California San Francisco; San Francisco, California 94518, United States
9. Chan Zuckerberg Biohub, San Francisco, California 94518, United States
10. Department of Health Systems Science, Kaiser Permanente Bernard J. Tyson School of Medicine; Pasadena, California 91101, United States

Contents of this supplement

Item	Title	Page
Table S1	Characteristics of the study cohort and individuals tested.	2
Table S2	Characteristics of individuals testing negative or positive for MPXV in the testing study, and those receiving mpox diagnoses.	4
Table S3	Assay results for positive specimens.	5
Table S4	Under-reporting estimates in sensitivity analyses limiting eligible specimens for analyses.	6
Table S5	Under-reporting estimates in sensitivity analyses with alternative natural history parameterization.	7
Table S6	Results of MPXV culture attempts and anti-MPXV seroconversion assessments in studies of individuals with subclinical infection.	8
Table S7	Cycle threshold values in relation to MPXV culture in clinical and non-clinical anorectal specimens.	9
Table S8	Reporting multipliers in analyses accounting for probabilities of MPXV culture and anti-MPXV seroconversion within the study cohort.	10
Table S9	Estimated effectiveness of JYNNEOS vaccination.	11
Table S10	Studies of MPXV infection prevalence or seroprevalence.	12
Table S11	Analytic basis for estimation of MSM population sizes for meta-analysis of surveillance studies.	13
Table S12	Pooled estimates of under-reporting multipliers across other studies.	14
Table S13	Symptoms among individuals testing positive for MPXV in molecular surveillance studies.	15
Table S14	Real-time PCR assay sequences.	16
Figure S1	Characteristics of the KPSC study cohort.	17
Figure S2	Modeled natural history of MPXV shedding in relation to date of reporting.	18
Figure S3	Association of cycle threshold value and infection stage with MPXV culture.	19
Figure S4	Estimated reporting multipliers according to alternative test specificity values.	20
Figure S5	Expected and observed MPXV infection prevalence or seroprevalence in other settings.	21
Figure S6	Risk adjustment for comparison of individuals recruited in STI clinic settings to the general MSM population.	22
Figure S7	Sensitivity of birth-death skyline model to sampling and transmission heterogeneity.	23
Figure S8	Under-reporting estimates applying alternative estimates of dispersion.	24
Figure S9	Power analysis.	25
Text S1	Supplemental references	26

Table S1: Characteristics of the study cohort and individuals tested.

Characteristic		Frequency, *n* (%)			Reweighted frequency[1]		
		Full cohort	Individuals tested	Individuals not tested	Full cohort	Individuals tested	Individuals not tested
		N=7,930	*N*=1,054	*N*=6,876			
Age group							
	16-25	996 (12.6)	70 (6.6)	926 (13.5)	12.5%	11.9%	12.6%
	26-35	3,310 (41.7)	460 (43.6)	2,850 (41.4)	41.9%	43.0%	41.7%
	36-45	2,714 (34.2)	407 (38.6)	2,307 (33.6)	33.9%	31.9%	34.2%
	≥46	910 (11.5)	117 (11.1)	793 (11.5)	11.7%	13.2%	11.5%
Race/ethnicity							
	White, non-Hispanic	2,244 (28.3)	319 (30.3)	1,925 (28.0)	28.1%	27.1%	28.3%
	Black, non-Hispanic	738 (9.3)	92 (8.7)	646 (9.4)	9.4%	10.1%	9.3%
	Asian/Pacific Islander, non-Hispanic	797 (10.1)	109 (10.3)	688 (10.0)	10.4%	12.5%	10.1%
	Hispanic, any race	3,340 (42.1)	417 (39.6)	2,923 (42.5)	42.0%	41.1%	42.1%
	Other/unknown	811 (10.2)	117 (11.1)	694 (10.1)	10.1%	9.2%	10.2%
Health insurance source							
	Commercial plan	5,677 (71.6)	760 (72.1)	4,917 (71.5)	71.4%	70.3%	71.6%
	Pre-paid/self-payment plan	1,071 (13.5)	162 (15.4)	909 (13.2)	13.4%	12.5%	13.5%
	Medicaid plan	854 (10.8)	85 (8.1)	769 (11.2)	11.1%	12.9%	10.8%
	Other/unknown	328 (4.1)	47 (4.5)	281 (4.1)	4.2%	4.4%	4.1%
Neighborhood deprivation index[2]							
	Quartile 1 (least deprived)	2,525 (31.8)	373 (35.4)	2,152 (31.3)	31.9%	32.4%	31.9%
	Quartile 2	1,817 (22.9)	227 (21.5)	1,590 (23.1)	23.1%	24.3%	22.9%
	Quartile 3	1,647 (20.8)	184 (17.5)	1,463 (21.3)	20.9%	21.4%	20.8%
	Quartile 4 (most deprived)	1,934 (24.4)	270 (25.6)	1,664 (24.2)	24.0%	21.8%	24.3%
	Unknown	7 (0.1)	0 (0.0)	7 (0.1)	0.1%	0.0%	0.1%
Prior-year healthcare utilization							
	<5 outpatient visits	2,086 (26.3)	174 (16.5)	1,912 (27.8)	26.4%	27.3%	26.3%
	5-10 outpatient visits	3,603 (45.4)	571 (54.2)	3,032 (44.1)	45.3%	44.1%	45.4%
	11-20 outpatient visits	1,691 (21.3)	238 (22.6)	1,453 (21.1)	21.3%	21.2%	21.3%
	≥21 outpatient visits	550 (6.9)	71 (6.7)	479 (7.0)	7.0%	7.4%	6.9%
	Any emergency department visit	1,376 (17.4)	171 (16.2)	1,205 (17.5)	17.2%	16.4%	17.4%
	Any inpatient admission	235 (3.0)	26 (2.5)	209 (3.0)	2.9%	2.3%	3.0%
Prior-year gonorrhea testing							
	0-1 tests	2,132 (26.9)	97 (9.2)	2,035 (26.9)	27.0%	27.9%	26.9%
	2-5 tests	4,967 (62.6)	767 (72.8)	4200 (61.1)	62.6%	62.3%	62.6%
	≥6 tests	831 (10.5)	190 (18.0)	641 (9.3)	10.4%	9.8%	10.5%
Gender and sexual orientation							
	Clinical record of sex with male partners[3]	5,077 (64.0)	815 (77.3)	4,262 (62.0)	63.3%	58.8%	64.0%
	Transgender identity[3]	268 (3.4)	6 (0.6)	262 (3.8)	3.7%	5.6%	3.4%
Sexual health measures							
	HIV pre-exposure prophylaxis	4,422 (55.8)	831 (78.8)	3,591 (52.2)	55.4%	53.1%	55.7%
	Doxycycline post-exposure prophylaxis	447 (5.6)	69 (6.5)	378 (5.5)	5.8%	6.8%	5.6%
	1 JYNNEOS dose	1,231 (15.5)	172 (16.3)	1,059 (15.4)	15.6%	16.2%	15.5%
	≥2 JYNNEOS doses	3,462 (43.7)	631 (59.9)	2,831 (41.2)	43.8%	45.0%	43.7%
History of sexually transmitted infections							
	HIV infection	1,435 (18.1)	165 (15.7)	1,270 (18.5)	18.6%	21.4%	18.1%
	Prior syphilis diagnosis	1,073 (13.5)	160 (15.2)	913 (13.3)	13.9%	16.0%	13.5%
	Prior-year gonorrhea: 1 diagnosis	965 (12.2)	202 (19.2)	763 (11.1)	12.5%	14.1%	12.2%
	Prior-year gonorrhea: ≥2 diagnoses	227 (2.9)	52 (4.9)	175 (2.5)	2.9%	2.8%	2.9%
	Prior-year chlamydia: 1 diagnosis	841 (10.6)	156 (14.8)	685 (10.0)	10.8%	12.0%	10.6%
	Prior-year chlamydia: ≥2 diagnoses	123 (1.6)	31 (2.9)	92 (1.3)	1.6%	1.8%	1.6%
	Other STI in prior year	21 (0.3)	1 (0.1)	20 (0.3)	0.2%	0.1%	0.3%
	Prior mpox diagnosis	9 (0.1)	2 (0.2)	7 (0.1)	0.1%	0.1%	0.1%
Other risk factors							

Alcohol or drug abuse diagnosis	821 (10.4)	93 (8.8)	728 (10.6)	10.3%	9.7%	10.4%

[1]We calculate stabilized weights via logistic regression with receipt of testing as the outcome variable to balance characteristics of individuals who did or did not receive testing.
[2]Neighborhood deprivation index is computed at the census-tract level according to a previously-described framework.[1]
[3]Variables are populated from electronic health records including notes from provider interactions and are not expected to identify all individuals with male sex partners or transgender identity. The study included only males with a history of anorectal testing for gonorrhea or chlamydia, an indication reserved for men who have sex with men.

Table S2: Characteristics of individuals testing negative or positive for MPXV in the testing study, and those receiving mpox diagnoses.

Characteristic		Frequency, *n* (%)		
		MPXV negative (testing study)	MPXV positive (testing study)	Diagnosed mpox cases
		N=1,048	*N*=6	*N*=15
Age group				
	16-25	70 (6.7)	0 (0.0)	1 (6.7)
	26-35	457 (43.6)	3 (50.0)	8 (53.3)
	36-45	405 (38.6)	2 (33.3)	5 (33.3)
	≥46	116 (11.1)	1 (16.7)	1 (6.7)
Race/ethnicity				
	White, non-Hispanic	316 (30.2)	3 (50.0)	2 (13.3)
	Black, non-Hispanic	92 (8.8)	0 (0.0)	1 (6.7)
	Asian/Pacific Islander, non-Hispanic	108 (10.3)	1 (16.7)	1 (6.7)
	Hispanic, any race	415 (39.6)	2 (33.3)	10 (66.7)
	Other/unknown	117 (11.2)	0 (0.0)	1 (6.7)
Health insurance source				
	Commercial plan	756 (72.1)	4 (66.7)	13 (86.7)
	Pre-paid plan	162 (15.5)	0 (0.0)	1 (6.7)
	Medicaid plan	85 (8.1)	0 (0.0)	1 (6.7)
	Other/unknown	45 (4.3)	2 (33.3)	0 (0.0)
Neighborhood deprivation index[1]				
	Quartile 1 (least deprived)	373 (35.6)	0 (0.0)	3 (20.0)
	Quartile 2	225 (21.5)	2 (33.3)	5 (33.3)
	Quartile 3	183 (17.5)	1 (16.7)	1 (6.7)
	Quartile 4 (most deprived)	267 (25.5)	3 (50.0)	6 (40.0)
Prior-year healthcare utilization				
	<5 outpatient visits	173 (16.5)	1 (16.7)	2 (13.3)
	5-10 outpatient visits	569 (54.3)	2 (33.3)	11 (73.3)
	11-20 outpatient visits	235 (22.4)	3 (50.0)	2 (13.3)
	≥21 outpatient visits	71 (6.8)	0 (0.0)	0 (0.0)
	Any emergency department visit	169 (16.1)	2 (33.3)	4 (26.7)
	Any inpatient admission	26 (2.5)	0 (0.0)	1 (6.7)
Prior-year gonorrhea testing				
	0-1 tests	96 (9.2)	1 (16.7)	1 (6.7)
	2-5 tests	764 (72.9)	3 (50.0)	13 (86.7)
	≥6 tests	188 (17.9)	2 (33.3)	1 (6.7)
Gender and sexual orientation				
	Clinical record of sex with male partners[2]	809 (77.2)	6 (100.0)	11 (73.3)
	Transgender identity	6 (0.6)	0 (0.0)	1 (6.7)
Sexual health measures				
	HIV pre-exposure prophylaxis	827 (78.9)	4 (66.7)	10 (66.7)
	Doxycycline post-exposure prophylaxis	68 (6.5)	1 (6.7)	2 (13.3)
	1 JYNNEOS dose	170 (16.2)	2 (33.3)	3 (20.0)
	≥2 JYNNEOS doses	628 (59.9)	3 (50.0)	4 (26.7)
History of sexually transmitted infections				
	HIV infection	164 (15.6)	1 (16.7)	3 (20.0)
	Prior syphilis diagnosis	158 (15.1)	2 (33.3)	4 (26.7)
	Prior-year gonorrhea: 1 diagnosis	200 (19.1)	2 (33.3)	3 (20.0)
	Prior-year gonorrhea: ≥2 diagnoses	52 (5.0)	0 (0.0)	1 (6.7)
	Prior-year chlamydia: 1 diagnosis	156 (14.9)	0 (0.0)	5 (33.3)
	Prior-year chlamydia: ≥2 diagnoses	31 (3.0)	0 (0.0)	0 (0.0)
	Other STI in prior year	1 (0.1)	0 (0.0)	0 (0.0)
	Prior mpox diagnosis	2 (0.2)	0 (0.0)	0 (0.0)
Other risk factors				
	Alcohol or drug abuse diagnosis	93 (8.9)	0 (0.0)	2 (13.3)

[1]Neighborhood deprivation index is computed at the census-tract level according to a previously-described framework.[1]

[2]Variables are populated from electronic health records including notes from provider interactions and are not expected to identify all individuals with male sex partners or transgender identity. The study included only males with a history of anorectal testing for gonorrhea or chlamydia, an indication reserved for men who have sex with men.

Table S3: Assay results for positive specimens.

Individual	Cycle threshold value	
	Mpox	Non-variola *Orthopoxvirus*
A	36.3	37.2
B	34.3	34.8
C	24.2	24.7
D	33.8	34.7
E	15.6	16.2
F (first specimen)	21.2	21.8
F (second specimen)	26.8	27.5

We present cycle threshold (c_T) values for quantitative polymerase chain reaction tests for mpox-specific and non-variola *Orthopoxvirus* probes among positive specimens; we provide forward and reverse primer and probe sequences in **Table S14**.

Table S4: Under-reporting estimates in sensitivity analyses limiting eligible specimens for analyses.

Estimate	Weighted estimates (95% confidence interval)[1]					
	MPXV infection prevalence (95% confidence interval), %		MPXV infection incidence per 100 person-years at risk		Under-reporting multiplier (95% confidence interval)	
	Clinically undetected and diagnosed cases	*Expected from diagnosed cases only*[2]	*Clinically undetected and diagnosed cases*	*Diagnosed cases only*	*Time-to-event analysis (primary)*	*Instantaneous prevalence comparison*[3]
All testing results included (*primary*)	0.91 (0.40, 1.63)	0.035%	22.2 (8.4, 48.0)	0.61	33.1 (16.2, 67.6)	26.3 (11.6, 46.5)
Excluding repeat positive results	0.86 (0.38, 1.57)	0.035%	21.1 (7.9, 46.3)	0.61	31.4 (15.2, 65.2)	25.1 (10.8, 45.4)
Excluding consecutive samples at <30-day intervals	0.88 (0.39, 1.59)	0.035%	21.5 (8.0, 46.9)	0.61	31.9 (15.4, 66.4)	25.3 (10.9, 45.5)

[1]We calculate stabilized weights via logistic regression with receipt of testing as the outcome variable to balance characteristics of individuals who did or did not receive testing (**Table S1**).
[2]We estimate expected prevalence, accounting for diagnosed cases alone, by projecting time series of the number of individuals shedding infection each day, based on sampled times of onset and cessation of shedding.
[3]We compare observed to expected prevalence throughout the study period according to the ratio $\beta = p/\bar{\pi}$.

Table S5: Under-reporting estimates in sensitivity analyses with alternative natural history parameterization.

Estimate	Weighted estimates (95% confidence interval)[1]	
	MPXV infection incidence, clinically undetected and diagnosed cases, per 100 person-years at risk	Under-reporting multiplier, time-to-event framework (95% confidence interval)
Parameterizing anorectal shedding duration from cross-sectional viral load measurements[1]	14.3 (5.5, 30.7)	21.3 (10.5, 42.9)
Parameterizing anorectal shedding duration from baseline and follow-up anorectal specimen testing[2]	34.7 (13.1, 75.2)	51.8 (25.2, 105.9)

[1]Estimates are based on a parameterization of time to loss of detectable anorectal shedding from a study reporting viral loads from anorectal specimens collected 1-20 days after symptoms onset.[2] We projected daily viral load distributions using a linear regression model fitted to the study data, and used these samples to define the cumulative distribution function for time to loss of detectable shedding.
[2]Estimates are based on a parameterization of time to loss of detectable anorectal shedding from a study[3] reporting results of MPXV detection in anorectal specimens collected at baseline and follow-up assessments, which occurred 1-8 and 21-32 days after symptoms onset, respectively.

Table S6: Results of MPXV culture attempts and anti-MPXV seroconversion assessments in studies of individuals with subclinical infection.

Study	Subject identifier	c_T in anorectal specimen	Outcome of viral culture	Seroconversion
Ogale et al., 2023[4]	B	17.2	Success	Not assessed
Ogale et al., 2023[4]	C	26.4	Success	Not assessed
De Baetselier et al., 2022[5]	1	26.7	Fail	Conversion
De Baetselier et al., 2022[5]	2	20.1	Success	Conversion
De Baetselier et al., 2022[5]	3	17.2	Success	Conversion
De Baetselier et al., 2022[5]	4	27.4	Fail	Not assessed
Agustí et al., 2024[6]	66	35.4	Fail	Not assessed
Agustí et al., 2024[6]	72	38.1	Success	Not assessed
Brosius et al., 2023[7]	DI_01	16.6	Success	Conversion
Brosius et al., 2023[7]	DI_02	20.6	Success	Conversion
Brosius et al., 2023[7]	DI_04	20.7	Success	Not assessed
Brosius et al., 2023[7]	DI_03	– –	– –	Conversion
Brosius et al., 2023[7]	DI_07	– –	– –	Conversion
Brosius et al., 2023[7]	PI_01	– –	– –	Conversion
Brosius et al., 2023[7]	PI_04	– –	– –	No conversion

Data are subset to individuals with positive MPXV detection by qPCR from each study, with c_T values, culture results, or seroconversion results reported at an individual level. An additional study[8] reporting viral culture from individuals with subclinical mpox was not included as individuals with subclinical infection did not test positive by qPCR from anal specimens. While seroconversion was not assessed longitudinally in the study by Ogale et al., both individuals with positive detections from anorectal specimens had elevated anti-MPXV IgM antibody levels at the time of their positive test.

Table S7: Cycle threshold values in relation to MPXV culture in clinical and non-clinical anorectal specimens.

Model	Case stratum	Slope parameter	$(c_T)_{50}$	Bayesian information criterion
Single slope and intercept, untransformed				
	All	–0.196	32.1	29.2
Single slope and intercept, log-transformed				
	All	–5.43	31.7	29.0
Single slope with differentiated intercepts, untransformed				
	Clinical	–0.198	32.4	
	Non-clinical	–0.198	30.7 (*p*=0.6)	33.0
Single slope with differentiated intercept, log-transformed				
	Clinical	–5.51	32.1	
	Non-clinical	–5.51	30.0 (*p*=0.7)	32.8
Differentiated slopes with single intercept, untransformed				
	Clinical	–0.195	32.4	
	Non-clinical	–0.205 (*p*=0.6)	30.9 (*p*=0.6)	33.1
Differentiated slopes with single intercept, log-transformed				
	Clinical	–5.47	32.1	
	Non-clinical	–5.58 (*p*=0.7)	30.0 (*p*=0.7)	32.8
Differentiated slopes and intercepts, untransformed				
	Clinical	–0.211	32.2	
	Non-clinical	–0.166 (*p*=0.4)	31.5 (*p*=0.6)	36.8
Differentiated slopes and intercepts, log-transformed				
	Clinical	–5.67	32.0	
	Non-clinical	–5.09 (*p*=0.5)	30.3 (*p*=0.6)	36.6

We estimate parameters of logistic models defining probabilities of successful viral culture according to functional forms following $1/\exp\left(-(\xi_0 + \xi_1 f(c_T)\right)$, comparing across those that differentiate slopes, intercepts, or both according to individuals' status as a diagnosed mpox case ("clinical") or as an individual sampled prospectively rather than on the basis of symptoms ("non-clinical"). Reported *p* values correspond to the two-sided hypothesis test for differences in the estimated parameter across case strata. Values in the column $(c_T)_{50}$ correspond to the c_T value associated with a 50% estimated probability of successful viral culture.

Table S8: Reporting multipliers in analyses accounting for probabilities of MPXV culture and anti-MPXV seroconversion within the study cohort.

Framework	Estimated prevalence (95% confidence interval)
Adjusted for individuals' probability of viable MPXV detection, given observed c_T value[1]	39.2 (17.0, 90.4)
Adjusted for the proportion of subclinical infections associated with seroconversion[2]	31.4 (13.6, 61.7)

[1]Estimates account for durations of culturable virus shedding consistent with the estimates presented in **Figure S3**, and associations of virus shedding with c_T values estimated via the best-fitting model (single slope and intercept, log-transformed c_T values) described in **Table S7**.
[2]Adjusted from primary estimate to account for the probability of seroconversion associated with subclinical infection (**Table S6**).

Table S9: Estimated effectiveness of JYNNEOS vaccination.

Measure	Framework	Description	Data for comparison groups	Estimate (95% confidence interval)
Vaccine effectiveness against diagnosed mpox, $\mathrm{VE}_D = (1 - \theta_S\theta_P) \times 100\%$	Case-control design	Compares adjusted odds of prior vaccination among individuals with diagnosed mpox (cases) versus individuals with gonorrhea (controls) via conditional logistic regression. Matching strata included individuals' HIV infection status and (among HIV-negative individuals) receipt or non-receipt HIV PrEP; receipt of doxycycline pre-exposure prophylaxis; and history of any syphilis diagnosis. Models further controlled for individuals' age group, receipt of *N. gonorrhea*/*C. trachomatis* testing in the prior year, and commercial or non-commercial insurance source (expected to proxy socioeconomic status) via covariate adjustment.	<u>Controls (*gonorrhea diagnosed*)</u>	
			Any vaccination: 135 (31.6%)	– –
			1 dose: 83 (19.4%)	– –
			2 doses: 209 (48.9%)	– –
			<u>Cases (*mpox diagnosed*)</u>	
			Any vaccination: 8 (53.3%)	71.5% (9.1%, 91.1%)
			1 dose: 3 (20.0%)	54.7% (–89.2%, 89.1%)
			2 doses: 4 (26.7%)	78.3% (16.8%, 94.3%)
	Negative control-corrected cohort design	Compares time to mpox diagnosis among JYNNEOS recipients to non-recipients, dividing the estimated adjusted hazard ratio by the adjusted estimated hazard ratio conveying the association of JYNNEOS vaccination with a negative-control outcome (gonorrhea), per Sanderson-Smith et al.[9] We estimated adjusted incidence rate ratios for each outcome via Poisson regression, controlling for HIV infection, receipt of HIV PrEP, receipt of doxycycline post-exposure prophylaxis, prior syphilis diagnosis, age group, prior-year gonorrhea testing, and commercial insurance source as covariates.	<u>VE against mpox (*negative control-uncorrected*)</u>	
			No vaccination: 0.8/100 PYAR	– –
			Any vaccination: 0.5/100 PYAR	59.6% (–17.9%, 86.1%)
			1 dose: 0.8/100 PYAR	29.1% (–176.5%, 81.7%)
			2 doses: 0.4/100 PYAR	69.6% (–6.1%, 91.4%)
			<u>VE against gonorrhea (*negative control outcome*)</u>	
			No vaccination: 20.8/100 PYAR	– –
			Any vaccination: 31.7/100 PYAR	–19.3% (–42.0%, –0.5%)
			1 dose: 30.1/100 PYAR	–16.9% (–47.3%, 7.2%)
			2 doses: 32.0/100 PYAR	–20.8% (–45.0%, –0.6%)
			<u>VE against mpox (*negative control-corrected*)</u>	
			Any vaccination	66.0% (–0.1%, 88.4%)
			1 dose	39.3% (–140.7%, 84.7%)
			2 doses	74.9% (10.8%, 92.9%)
Vaccine effectiveness against MPXV infection, $\mathrm{VE}_S = (1 - \theta_S) \times 100\%$	Matched test-negative design	Compares adjusted odds of prior vaccination among individuals testing positive for MPXV infection in anorectal specimens (cases) versus individuals testing negative (controls). Matching strata included individuals' HIV infection status and (among HIV-negative individuals) receipt or non-receipt HIV PrEP; receipt of doxycycline pre-exposure prophylaxis; and history of any syphilis diagnosis.	<u>Controls (*testing negative*)</u>	
			No vaccination: 274 (23.2%)	– –
			Any vaccination: 909 (76.8%)	– –
			1 dose: 184 (15.6%)	– –
			2 doses: 725 (61.3%)	– –
			<u>Cases (*testing positive*)</u>	
			No vaccination: 1 (14.3%)	– –
			Any vaccination: 6 (85.7%)	52.6% (–166.9%, 91.6%)
			1 dose: 2 (28.6%)	41.4% (–432.1%, 93.6%)
			2 doses: 4 (57.1%)	58.8% (–157.6%, 93.4%)
	Cohort design	Compares weighted incidence rates of MPXV infection among vaccinated individuals to unvaccinated individuals, corrected for estimated degree of under-reporting within each stratum.	No vaccination: 35.8/100 PYAR	– –
			Any vaccination: 11.7/100 PYAR	50.7% (–91.8%, 94.5%)
Vaccine effectiveness against progression to diagnosed mpox, given MPXV infection, $\mathrm{VE}_P = (1 - \theta_P) \times 100\%$	Model-based estimate	Obtained numerically, defining the risk (hazard, rate, …) ratio of diagnosed mpox associated with vaccination as the product of the risk (hazard, rate, …) ratio of infection, given vaccination, times the risk (hazard, rate, …) ratio of progression to disease, given infection, associated with vaccination.	– –	41.0% (–244.6%, 89.9%)

PYAR: Person-year at risk.

Table S10: Studies of MPXV infection prevalence or seroprevalence.

Study type	Location	Citation[1]	Population sampled	Dates	Observed prevalence[2]	Expected prevalence (95% confidence interval)
Molecular testing						
	Paris, France	Ferré et al., 2022[10]	MSM living with HIV or taking HIV pre-exposure prophylaxis and receiving routine screening for *C. trachomatis* and *N. gonorrhoeae*, with no suspicion of mpox illness	June 5 to July 11, 2022	6.5% (13/200)	0.088% (0.036–0.163%)
	Antwerp, Belgium	De Baetselier et al., 2022[5] and van Dijck, et al.[11]	MSM living with HIV or taking HIV pre-exposure prophylaxis and receiving routine screening for *C. trachomatis* and *N. gonorrhoeae*, with no suspicion of mpox illness			
				May 1-30, 2022	1.8% (4/224)	0.005% (0.002–0.009%)
				June 1-30, 2022	4.8% (7/146)	0.017% (0.009–0.025%)
				July 1 to 30 September, 2022	3.9% (7/181)	0.012% (0.001–0.028%)
	Geneva and Zürich, Switzerland	Hampel et al., 2023[12]	MSM taking HIV pre-exposure prophylaxis and receiving routine screening for *C. trachomatis* and *N. gonorrhoeae*, with no suspicion of mpox illness	August 23 to October 4, 2022	1.0% (2/201)	0.006% (0.001–0.015%)
	Washington, DC, United States	Ogale et al., 2023[4]	Individuals presenting for their first JYNNEOS vaccine dose, with no suspicion of mpox illness	August 11-31, 2022		0.031% (0.017–0.050%)
			With restriction to anorectal specimens		1.2% (2/164)	
			Without restriction to anorectal specimens		1.8% (3/164)	
	Barcelona, Spain	Agustí et al., 2024[6]	MSM recruited at a community center with no suspicion of mpox illness	August 1 to October 31, 2022		0.024% (0.001–0.091%)
			With restriction to anorectal specimens		1.8% (2/112)	
			Without restriction to anorectal specimens		6.2% (7/113)	
Serological testing						
	San Francisco, United States	Minhaj et al., 2023[13]	MSM receiving services at sexual health clinics with no suspicion of mpox illness	June 28 to August 26, 2022	8.0% (18/225)	1.02% (0.96–1.09%)
	New York, United States	Pathela et al., 2024[14]	MSM receiving routine syphilis and HIV testing, with no history of receipt of vaccina virus-based vaccines and with no suspicion of prior mpox illness or exposure	July 7 to September 1, 2022	7.8% (16/166)	1.32% (1.09–1.63%)
	Rome, Italy	Matusali et al., 2023[15]	Men presenting for their first JYNNEOS vaccine dose, with no history of receipt of vaccinia virus-based vaccines and with no prior mpox diagnosis	August 8 to September 9, 2022	12.8% (18/141)	0.19% (0.16–0.23%)
	Washington, DC, United States	Ogale et al., 2023[4]	Individuals presenting for their first JYNNEOS vaccine dose, with no suspicion of mpox illness (limited to individuals providing anorectal swab)	August 11-31, 2022	14.5% (47/324)	1.17% (0.97–1.44%)
	Berlin, Germany	Marcus et al., 2024[16]	MSM recruited in private practices and community-based checkpoints specialized in care for HIV and sexually-transmitted infections	April 1 to June 30, 2023	16.4% (149/908)	2.36% (1.95–2.90%)

[1]We identified studies through a systematic review including a PubMed search augmented by forward- and back-tracking of citations.
[2]Analyses restricted results from molecular testing studies to those generated from anorectal specimens. We restricted results from serological testing studies to those enrolling unvaccinated or previously uninfected individuals (for studies during early phases of the outbreak in summer, 2022), or those using an antibody marker that distinguishes natural infection from vaccine response (for studies undertaken after vaccine implementation).

Table S11: Analytic basis for estimation of MSM population sizes for meta-analysis of surveillance studies.

Setting	Estimate (95% CI)	Description
Île-de-France (Paris region), France	95,661 (58,240–133,087)	A previous study[17] estimated the population of MSM within Île-de-France as of 2014 (*N*=93,178). We scaled this estimate by the population size of Île-de-France in 2014 and extrapolated to the population as of 2022.
Belgium	111,620 (92,413–130,811)	Two prior estimates[18,19] of the population of MSM within Belgium were available (*N*=106,336 in 2013 and *N*=144,753 in 2015). We scaled these estimates by the population sizes of Belgium as of 2013 and 2015 and extrapolated to the population as of 2022. We multiplied by the population size of Belgium as of 2022 and assumed a normal distribution in pooling the estimates.
Lazio, Italy	128,865 (105,388–152,348)	Direct estimates of the population size of MSM in Lazio or Rome, Italy were not available. A previous study[20] estimated that MSM accounted for 2.7% of the adult male population in Verona, Italy as of 2014 (*N*=10,952); we scaled this estimate by the size of the population of men aged 16-64 years in Verona and multiplied by the size of the population of adult men aged 16-64 in the metropolitan city of Rome to obtain the urban MSM population. We added 1% of the population size of men aged 16-64 within the remaining administrative districts within Lazio to account for MSM outside the urban core, assuming a lower proportion in such regions would be MSM. We assumed the same ratio of the mean to standard deviation as reported in Verona and assumed a normal distribution.
Switzerland	88,758 (70,986–106,535)	A previous study[21] estimated the population of MSM within Switzerland as of 2011 (*N*=80,000). We scaled this estimate by the population size of Switzerland as of 2011, and multiplied by the population size of Switzerland as of 2022. We assumed a normal distribution.
Berlin, Germany	94,931 (76,312 - 113,496)	A previous study[22] estimated the population of MSM within Berlin as of 2008 (*N*=91,000). We scaled this estimate by the population size of Berlin in 2008 and extrapolated to the population as of 2024. Measures of uncertainty were not provided with the original report. We therefore defined standard deviation in the estimate as being equal to 10% of the mean and assumed a normal distribution.
Barcelona, Spain	42,613 (34,221 - 50,980)	A precious study[23] estimated the population of MSM comprised 5.3% of Barcelona's male population in 2012. Assuming this proportion remains constant through 2024, we extrapolated to the male population as of 2024. Measures of uncertainty were not provided with the original report. We therefore defined standard deviation in the estimate as being equal to 10% of the mean and assumed a normal distribution.
Washington, DC, United States	34,857 (28,840–42,895)	A previous study[24] estimated the population of MSM within Washington, DC as of 2016 (*N*=35,867). We scaled this estimate by the population size of Washington, DC in 2016 and extrapolated to the population as of 2022. Measures of uncertainty were not provided with the original report. We therefore defined standard deviation in the estimate as being equal to 10% of the mean and assumed a normal distribution.
San Francisco, California, United States	64,665 (60,607–68,730)	A previous study[25] estimated the population size of MSM within San Francisco as of 2017 (*N*=69,974). We scaled this estimate by the population size of San Francisco in 2017 and extrapolated to the population as of 2022. We assumed a normal distribution for reported uncertainty bounds and for our projection.
New York, New York, United States	218,375 (175,551–261,181)	A previous study[24] estimated the population size of MSM within New York City as of 2016 (*N*=224,020). We scaled this estimate by the population size of New York City in 2016 extrapolated to the population as of 2022. Measures of uncertainty were not provided with the original report. We therefore defined standard deviation in the estimate as being equal to 10% of the mean and assumed a normal distribution.

Table S12: Pooled estimates of under-reporting multipliers across other studies.

Studies included	Estimate (95% confidence interval)[1]	
	Without risk adjustment	With risk adjustment
All molecular testing studies	189 (90.7, 394)	136 (65.1, 283)
All serological testing studies	12.9 (5.59, 29.7)	9.24 (4.01, 21.3)
All molecular and serological testing studies	54.5 (20.7, 143)	39.1 (14.9, 103)

[1]Pooled estimates are generated via inverse variance-weighted random effects models including under-reporting multipliers from each selection of studies.

Table S13: Symptoms among individuals testing positive for MPXV in molecular surveillance studies.

Reference	Setting and design	Outcomes[1]	
		Individuals followed	Individuals with symptoms or mpox diagnosis
Agustí et al., *Nat Comm* 2024[6]	Testing via self-collected oropharyngeal and anorectal specimens with telephone follow-up 21 days after any positive test for symptom interview	7	2
de Batselier et al., *Nat Med* 2022;[5] van Dijck et al., *Lancet Microbe* 2023[11]	Testing via oropharyngeal and anorectal specimens with accompanying symptom interview and clinical assessment, with follow-up 21-37 days after testing	18	5
Hampel et al., *Lancet Microbe* 2023[12]	Testing via oropharyngeal and anorectal specimens within an existing longitudinal cohort study collecting symptoms data via web application	2	1
Ferré et al., *Ann Intern Med* 2022[10]	Testing via anorectal specimens with subsequent outreach to individuals testing positive to collect symptoms data	13	2
Ogale et al., *Clin Infect Dis* 2024[4]	Testing via anorectal and pharyngeal specimens within a cohort study involving 3 clinic visits over two months with symptoms interview at study visits.	3	1
Kaiser Permanente Southern California cohort (current study)	Testing of anorectal specimens collected for STI screening, with monitoring for mpox-related healthcare utilization among cohort members in integrated healthcare system	6	0
**Total**		49	11

[1]Data encompass all individuals reported to experience infection, as detected by either oropharyngeal or anorectal specimens (a broader population than those included in analyses as reported in **Table S10**). Studies differed in monitoring for symptoms based on active or passive surveillance modalities.

Table S14: Real-time PCR assay sequences.

Assay	Primer/probe	Sequence
Non-variola *Orthopoxvirus*		
	Forward	5'-TCA ACT GAA AAG GCC ATC TAT mGA-3'
	Reverse	5'-GAG TAT AGA GCA CTA TTT CTA AAT CCmC A-3'
	Probe	5' - /5YakYel/CCA TGC AAT/ZEN ATA CGT ACA AGA TAG TAG CCA AC / 3IABkFQ/-3'
Mpox		
	Forward	5'-ACG TGT TAA ACA ATG GGT GAmU G-3'
	Reverse	5'-AAC ATT TCC ATG AAT CGT AGT mCC-3'
	Probe	5'-/56-FAM/TGA ATG AAT /ZEN/ GCG ATA CTG TAT GTG TGG G/3IABkFG/-3'

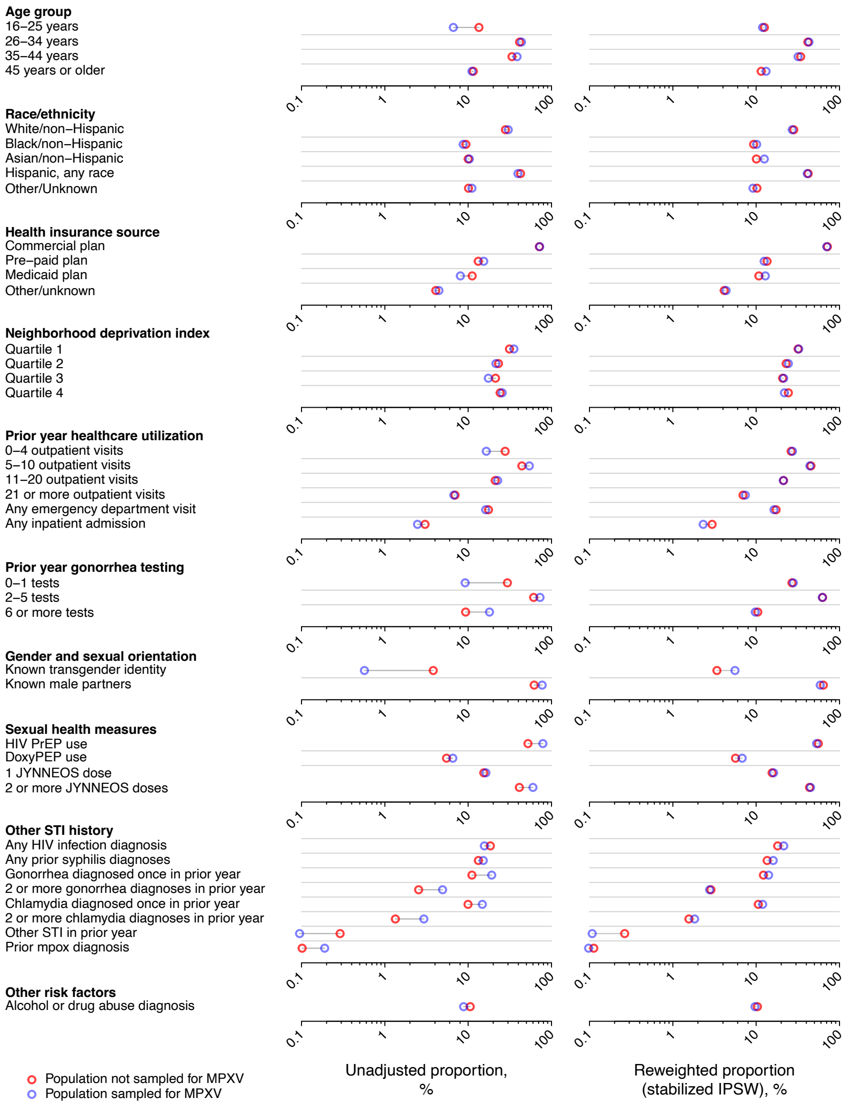

Figure S1: Characteristics of the KPSC study cohort. We present distributions of demographic and clinical characteristics within the (left) unweighted and (right) reweighted study cohort, with weights estimated by logistic regression to balance characteristics of individuals who received or did not receive MPXV testing by anorectal swabs within the analysis cohort (*N*=7,930 individuals). We present underlying data in **Table S1** and compare individuals testing positive or negative for MPXV, in the unweighted and weighted samples, in **Table S2**.

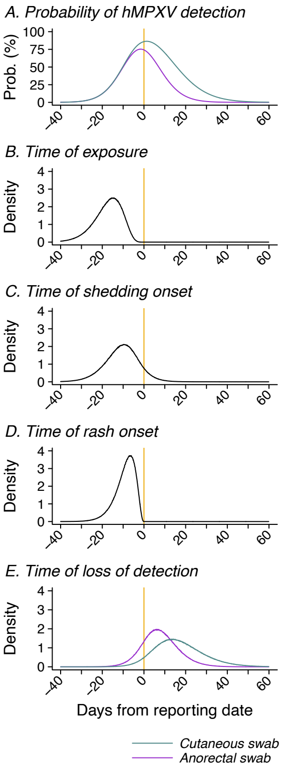

Figure S2: Modeled natural history of MPXV shedding in relation to date of reporting. We present (**A**) daily probabilities of MPXV detection by PCR in cutaneous and anorectal swabs, along with (**B**-**E**) fitted distributions for times-to-event for underlying epidemiologic parameters including times from exposure, onset of MPXV shedding, onset of clinical rash, and time to cessation of shedding detectable by PCR.

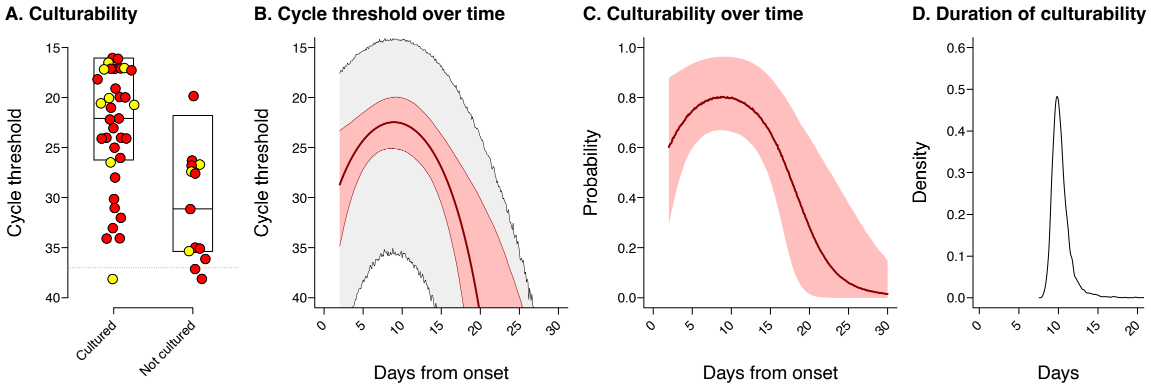

Figure S3: Association of cycle threshold value and infection stage with MPXV culture. We illustrate (**A**) c_T values in anorectal specimens from individuals with successful or unsuccessful viral culture results (*N*=48), colored according to their status as symptomatic individuals diagnosed as mpox cases (red) or individuals enrolled in symptoms-agnostic prospective studies (yellow; **Table S7**). Next, we illustrate (**B**) point estimates (red solid line), 95% confidence intervals (pink shaded area), and 95% prediction intervals (grey shaded area) for MPXV c_T values in anorectal specimens as a function of time from symptoms onset, alongside accompanying probabilities of successful viral culture from these specimens (**C**) based on the best-fitting model (**Table S7**). The solid red line and pink shaded area indicate point estimates and accompanying 95% confidence intervals, respectively. Last, we illustrate (**D**) the fitted distribution of individuals' total duration of culturable virus shedding, defined as beginning at the time of shedding onset.

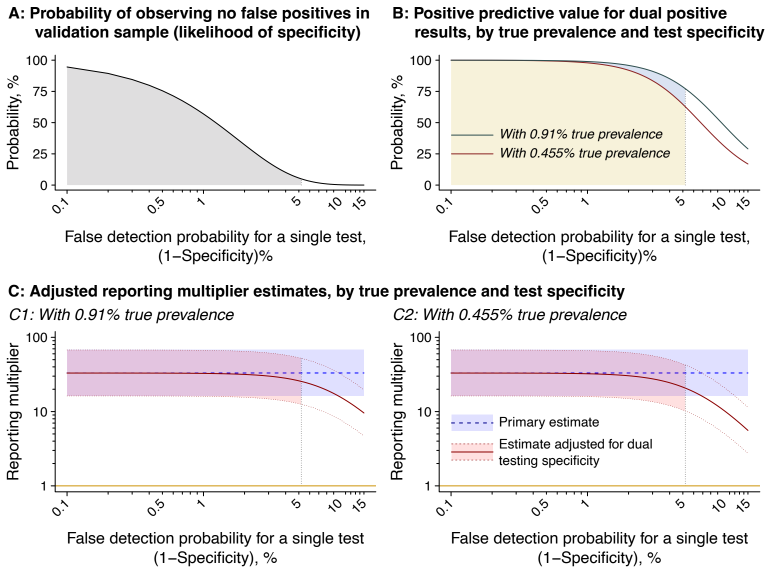

Figure S4: Estimated reporting multipliers according to alternative test specificity values. We present (**A**) the likelihood of the indicated single-test specificity values, given the observation of 0 false positives among 64 true negative specimens in an external assessment of the study assay.[26] We compute this value via a binomial distribution with x=0, n=64, and p equal to the false detection probability for a single test (1–Specificity). The shaded area (for panel **A** and all subsequent panels) delineates the range of single-test false detection probabilities for which the probability of observing x=0 false positive results among n=64 specimens exceeds 95%. Next, we illustrate (**B**) estimates of positive predictive value for dual testing with true infection prevalence equal to 0.91% (as estimated in the study involving n=1,190 specimens; blue) and 0.455% (half the estimated value; brown). Below, we illustrate (**C**) reporting multipliers for adjusted for potential false positives under the dual testing procedure. Red shaded areas delineate 95% confidence intervals around point estimates (center line; red). We illustrate primary estimates without specificity adjustment in blue (shaded area, 95% confidence interval; point estimate, dotted line). Panel A1 (left) illustrates reporting multipliers under an assumed true prevalence of 0.91%, and panel A2 (right) illustrates reporting multipliers under an assumed true prevalence of 0.455%.

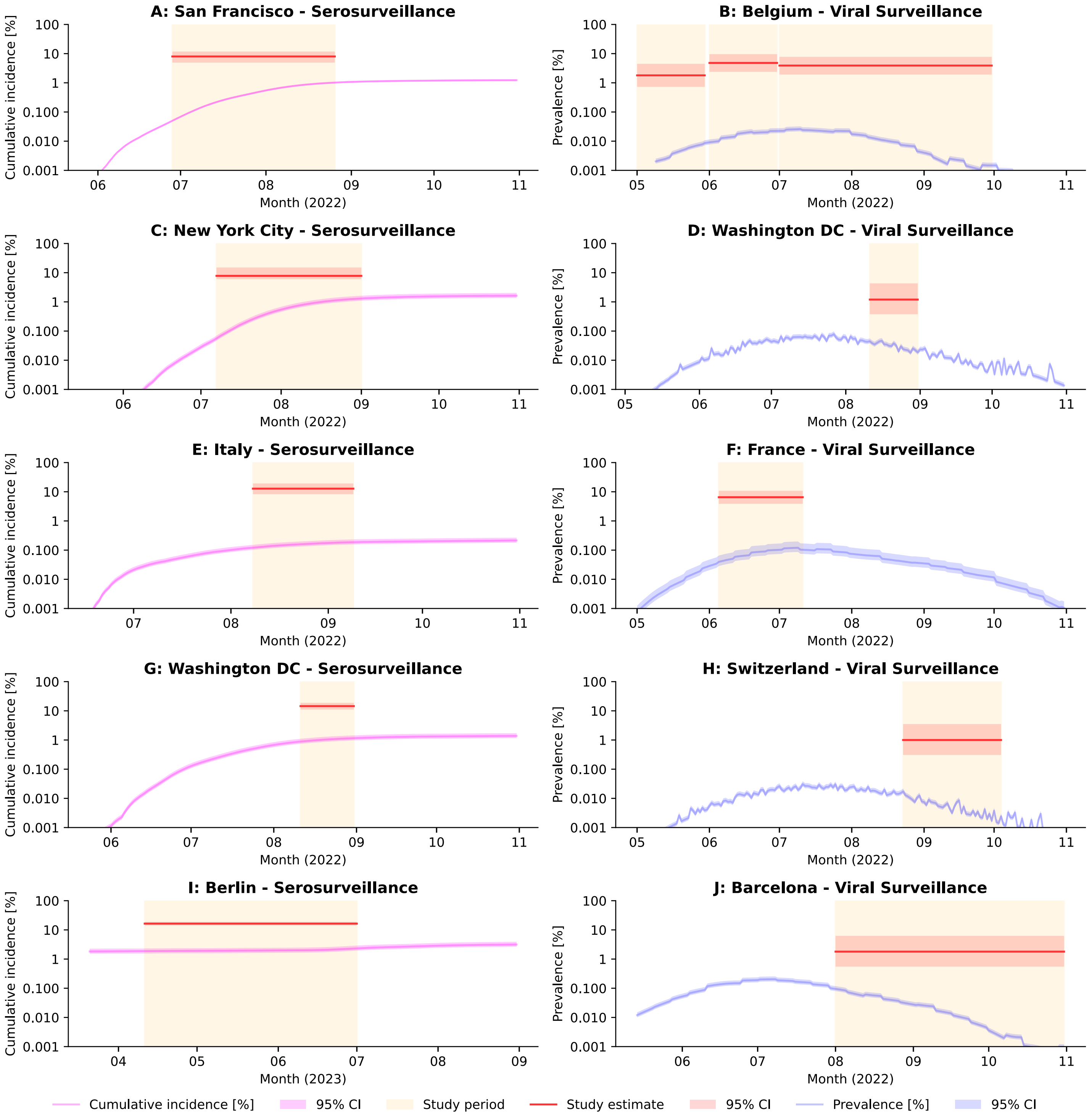

Figure S5: Expected and observed MPXV infection prevalence or seroprevalence in other settings. We illustrate estimated seroprevalence (left column; pink line, with shaded area delineating 95% confidence intervals) and infection prevalence (right column; blue line, with shaded area delineating 95% confidence interval) based on reported cases within each setting (**Table S10**; **Table S11**). Tan shaded areas delineate the study periods, with red lines denoting observations within each study (shaded areas delineate 95% confidence intervals around studies' prevalence or seroprevalence estimates).

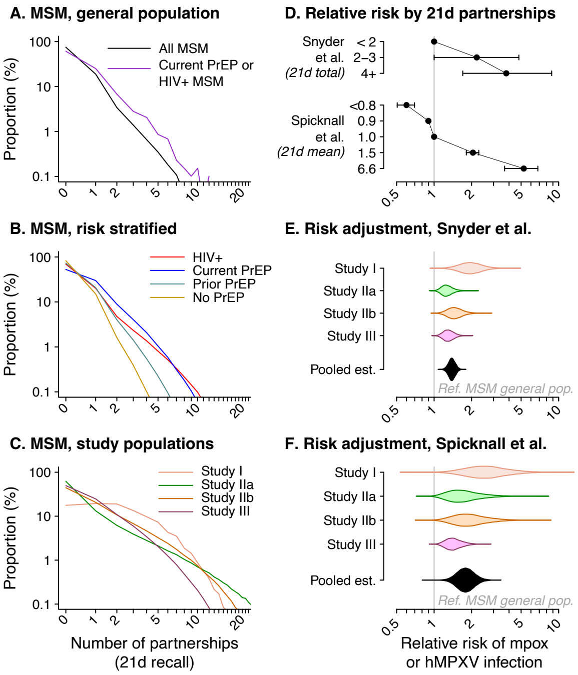

Figure S6: Risk adjustment for comparison of individuals recruited in STI clinic settings to the general MSM population. We present comparisons of partnership counts within a 21-day recall period among differing MSM strata within a general population sample (**A**, **B**), as reported in the ARTnet study (*N*=4904),[27,28] along with the strongly right-tailed distribution of partnership counts within the same recall period among individuals recruited in sexual health facilities (**C**) based on three studies.[29–31] In the right-hand column, we illustrate reported associations of number of unprotected anal intercourse partnerships within a 21-day recall period with mpox risk based on a case-control study[32] and modeling study[33] (**D**). Below, we illustrate estimates of the relative risk of mpox among individuals recruited in sexual health facilities versus the general MSM population, for the individual studies presented in (**C**) and pooled across these studies, using relative risk parameters based on the case-control study (**E**) and the modeling study (**F**).

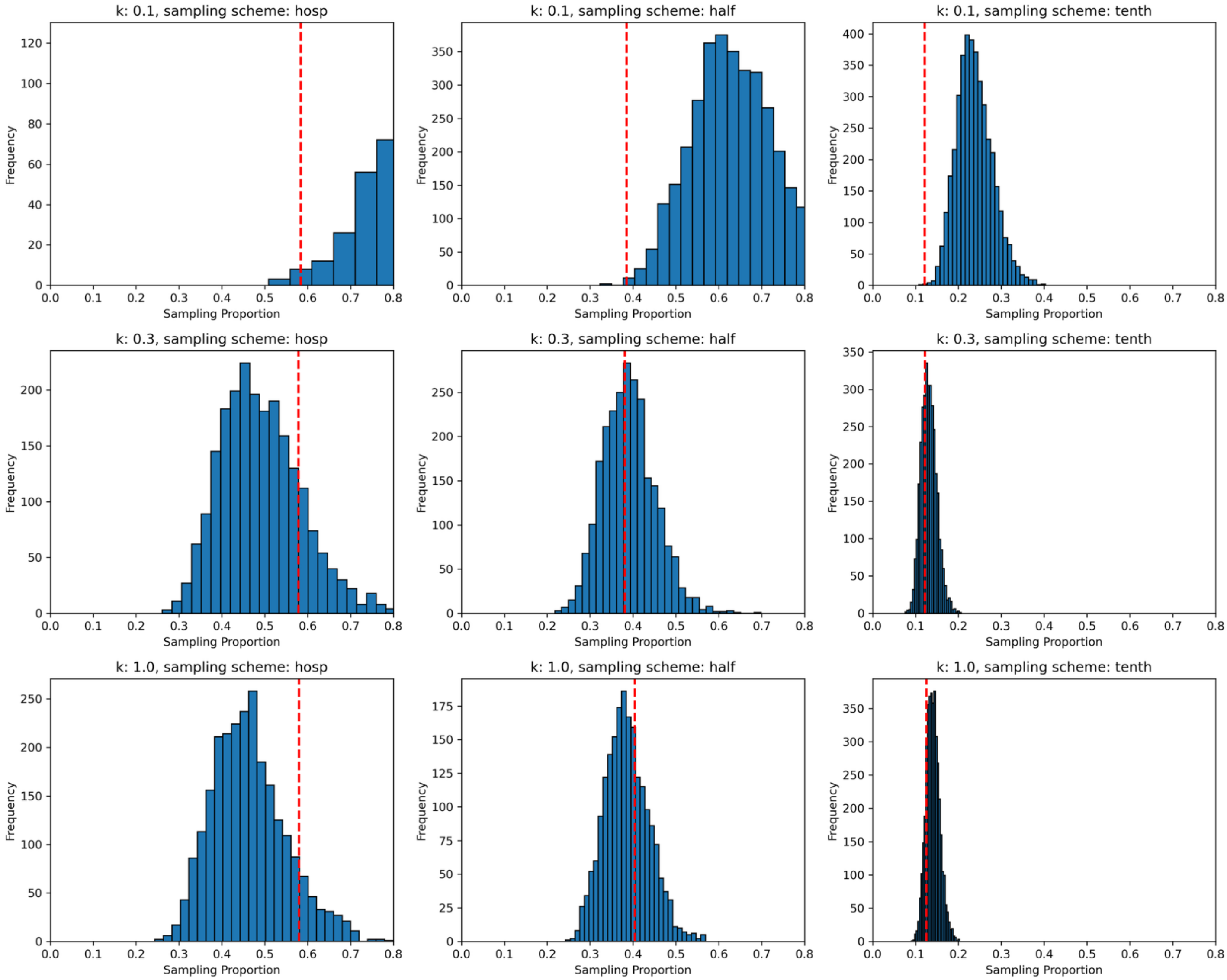

Figure S7: Sensitivity of birth-death skyline model to sampling and transmission heterogeneity. We illustrate the ability of our birth-death skyline model to accurately capture the true sampling proportions by simulating MPXV transmission dynamics with different values of the dispersion parameter (*k*=0.1, 0.3, 1.0) and sampling schemes. Under the sampling scheme "hosp", time to sampling is defined as the estimated time to present to healthcare in the UK in 2022;[34] the "half" and "tenth" sampling schemes reduce this rate by 50% and 90%, respectively. Histogram bars indicate the distribution of sampling proportion estimates from analyses of simulated data, while red dashed lines represent the true sampling proportion used to parameterize simulations. The center row of panels corresponds to $k = 0.3$, consistent with prior empirical findings,[35–37] and demonstrates the best alignment between true and estimated values of *p* under each sampling scheme.

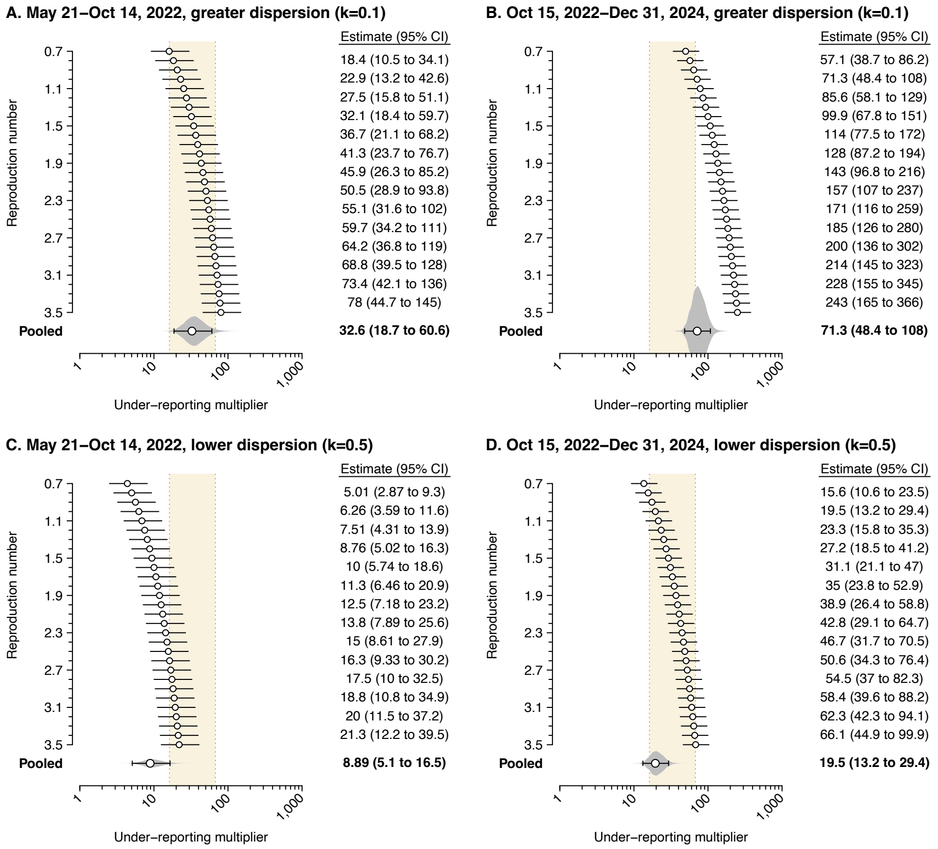

Fig. S8: Under-reporting estimates applying alternative estimates of dispersion. We illustrate under-reporting multipliers based on a comparison of diagnosed cases to the estimated population size of MPXV infections in Los Angeles County from phylogenetic analyses of sequenced cases, accounting for introductions from external geographies, for the periods from May 21 to October 14, 2022 (*N*=271 isolates) and from October 15 to December 24, 2022 (*N*=226 isolates). Within each panel, estimates are conditioned on the reproduction number, *R*. We illustrate the probability density of mean daily *R* values throughout the analysis period in purple. Pooled estimates presented in bold at the bottom of the figure are weighted according to the period-specific probability density of *R*. Top panels (**A**, **B**) present estimates under a scenario with extreme dispersion in the offspring distribution ($k = 0.1$), while bottom panels (**C**, **D**) consider a scenario with lower dispersion ($k = 0.5$).

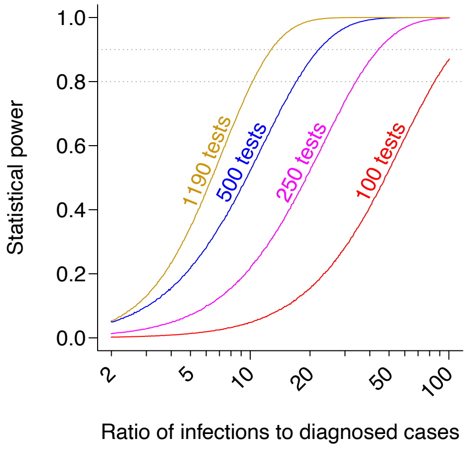

Fig. S9: Power analysis. We illustrate statistical power—defined as the probability of correctly rejecting the null hypothesis when it is false—at two-sided $p<0.05$ for studies testing 100 (red), 250 (pink), 500 (blue), or 1190 (gold) anorectal specimens for MPXV infection by PCR, under scenarios where the ratio of true to expected prevalence under the null hypothesis (0.035%, per **Table S4**) ranges from 2-fold to 100-fold higher. Dotted grey lines indicate thresholds at which power exceeds 80% and 90% for each sample size.

SUPPLEMENTAL REFERENCES

1. Messer, L. C. *et al.* The development of a standardized neighborhood deprivation index. *J Urban Health* **83**, 1041–62 (2006).

2. Yang, Y. *et al.* Longitudinal viral shedding and antibody response characteristics of men with acute infection of monkeypox virus: a prospective cohort study. *Nat Comm* **15**, 4488 (2024).

3. Meyerowitz, E. A. *et al*. Anorectal testing for mpox virus infection in men who have sex with men with and without proctitis. *Clin Infect Dis* **76**, 934–7 (2023).

4. Ogale, Y. P. *et al.* Evidence of mpox virus infection among persons without characteristic lesions or rash presenting for first dose of JYNNEOS vaccine—District of Columbia, August 2022. *Clin Infect Dis* **77**, 298–302 (2023).

5. De Baetselier, I. *et al*. Retrospective detection of asymptomatic monkeypox virus infections among male sexual health clinic attendees in Belgium. *Nat Med* **28**, 2288–92 (2022).

6. Agustí, C. *et al*. Self-sampling monkeypox virus testing in high-risk populations, asymptomatic or with unrecognized Mpox, in Spain. *Nat Commun* **14**, 5998 (2023).

7. Brosius, I. *et al.* Presymptomatic viral shedding in high-risk mpox contacts: a prospective cohort study. *J Med Virol* **95**, e28769 (2023).

8. Moschese, D. *et al*. Isolation of viable monkeypox virus from anal and urethral swabs, Italy, May to July, 2022. *Eurosurveillance* **27**, 2200675 (2022).

9. Sanderson, E., Macdonald-Wallis, C., Davey Smith, G. Negative control exposure studies in the presence of measurement error: implications for attempted effect estimate calibration. *Int J Epidemiol* **47**, 587–96 (2018).

10. Ferré, V. M., *et al.* Detection of monkeypox virus in anorectal swabs from asymptomatic men who have sex with men in a sexually transmitted infection screening program in Paris, France. *Ann Intern Med* **175**, 1491–2 (2022).

11. Dijck, C. V., *et al.* Mpox screening in high-risk populations finds no asymptomatic cases. *Lancet Microbe*, **4**, e132–3 (2023).

12. Hampel, B. *et al*. Low prevalence of asymptomatic mpox in populations at high risk. *Lancet Microbe* **4**, e856 (2023).

13. Minhaj, F. S. *et al.* Prevalence of undiagnosed monkeypox virus infections during global mpox outbreak, United States, June–September, 2022. *Emerg Infect Dis* **29**, 2307–14 (2023).

14. Pathela, P. *et al.* Serological evidence of mpox virus infection during peak mpox transmission in New York City, July to August, 2022. *J Infect Dis* **230**, jiae181 (2024).

15. Matusali, G. *et al.* Asymptomatic mpox virus infection in subjects presenting for MVA-BN vaccine. *Clin Infect Dis* **77**, 1483–4 (2023).

16. Marcus, U. *et al.* A seroprevalence study indicates a high proportion of clinically undiagnosed MPXV infections in men who have sex with men in Berlin, Germany. *BMC Infect Dis* **24**, 1153 (2024).

17. Marty, L. et al. Revealing geographical and population heterogeneity in HIV incidence, undiagnosed HIV prevalence and time to diagnosis to improve prevention and care: estimates for France. *J Int AIDS Soc* **21**, e25100 (2018).

18. Marcus, U., Hickson, F., Weatherburn, P. & Schmidt, A. J. Estimating the size of the MSM populations for 38 European countries by calculating the survey-surveillance discrepancies (SSD) between self-reported new HIV diagnoses from the European MSM internet survey (EMIS) and surveillance-reported HIV diagnoses among MSM in 2009. *BMC Public Health* **13**, 919 (2013).

19. Marty, L. et al. Estimates of the HIV undiagnosed population in Belgium reveals higher prevalence for MSM with foreign nationality and for geographic areas hosting big cities. *J Int AIDS Soc* **22**, e25371 4(2019).

20. Johnston, L. G. et al. Populations size estimations using SS-PSE among MSM in four European cities: how many MSM are living with HIV? *Eur J Public Health* **31**, 1129–36 (2021).

21. Schmidt, A. J. & Altpeter, E. The Denominator problem: estimating the size of local populations of men-who-have-sex-with-men and rates of HIV and other STIs in Switzerland. *Sex Transm Infect* **95**, 285–291 (2019).

22. Marcus, U., Schmidt, A. J., Hamouda, O. & Bochow, M. Estimating the regional distribution of men who have sex with men (MSM) based on Internet surveys. *BMC Public Health* **9**, 180 (2009).

23. Martí-Pastor, M. *et al.* Epidemiology of infections by HIV, syphilis, gonorrhea and *Lymphogranuloma venereum* in Barcelona City: a population-based incidence study. *BMC Public Health* **15**, 1015 (2015).

24. Grey, J. A. *et al.* Estimating the population sizes of men who have sex with men in US states and counties using data from the American Community Survey. *JMIR Public Health Surveill* **2**, e14 (2016).

25. Raymond, H. F., McFarland, W. & Wesson, P. Estimated population size of men who have sex with men, San Francisco, 2017. *AIDS Behav* **23**, 1576–9 (2019).

26. Nörz, D. *et al*. Rapid adaptation of established high-throughput molecular testing infrastructure for monkeypox virus detection. *Emerg Infect Dis* **28**, 1765–1769 (2022).

27. Mann, L. M., *et al*. Correlations between community-level HIV preexposure prophylaxis coverage and individual-level sexual behaviors among United States MSM. *AIDS* **36**, 2015–23 (2022).

28. Weiss, K. M., *et al*. Egocentric sexual networks of men who have sex with men in the United States: results from the ARTnet study. *Epidemics* **30**, 100386 (2020).

29. Montaño, M. A. *et al.* Differences in sexually transmitted infection risk comparing preexposure prophylaxis users and propensity score matched historical controls in a clinic setting. *AIDS* **33**, 1773–80 (2019).

30. Montaño, M. A. *et al.* Changes in sexual behavior and STI diagnoses among MSM initiating PrEP in a clinic setting. *AIDS Behav* **23**, 548–55 (2019).

31. DeVost, M. A., Beymer, M. R., Weiss, R. E., Shover, C. L., Bolan, R. K. App-based sexual partner seeking and sexually transmitted infection outcomes: a cross-sectional study of HIV-negative men who have sex with men attending a sexually transmitted infection clinic in Los Angeles, California. *Sex Transm Dis* **45**, 394–9 (2018).

32. Snyder, R. E. et al. Sexual exposures associated with mpox infection: California, November, 2022 to June, 2023. *J Infect Dis* **229**, S188–96 (2024).

33. Spicknall IH. Modeling the impact of sexual networks in the transmission of monkeypox virus among gay, bisexual, and other men who have sex with men — United States, 2022. *Morb Mortal Wkly Rep* **71**, 1131–5 (2022).

34. Hinch, R. *et al.* Quantification of the time-varying epidemic growth rate and of the delays between symptom onset and presenting to healthcare for the mpox epidemic in the UK in 2022. *Sci Rep* **14**, 19755 (2024).

35. Paredes, M. I., *et al*. Underdetected dispersal and extensive local transmission drove the 2022 mpox epidemic. *Cell* **187**,1374-1386.e13 (2024).

36. Paredes, M. I. *et al.* Viral introductions and return to baseline sexual behaviors maintain low-level mpox incidence in Los Angeles County, USA, 2023–2024. *medRxiv* [Preprint] (2025) doi: 10.1101/2025.03.14.25323999.

37. Maniscalco D, Robineau O, Boëlle PY, Mazzoli M, Barret AS, Chazelle E, et al. Adaptive behavior in response to the 2022 mpox epidemic in the Paris region. medRxiv [Preprint] (2024) doi:10.1101/2024.10.25.24315987v1.
