# Supplementary material for: Extensive cryptic circulation sustains mpox among men who have sex with men
Source: Nat Commun. 2026 May 13;17:4198. doi: 10.1038/s41467-026-72749-2 (PMC13172365; doi:10.1038/s41467-026-72749-2)
Supplement: Supplementary file 2 — Reporting Summary [file 41467_2026_72749_MOESM2_ESM.pdf]

nature portfolio

Corresponding author(s): Lewnard, Joseph

Last updated by author(s): 21 October 2025

Reporting Summary

Nature Portfolio wishes to improve the reproducibility of the work that we publish. This form provides structure for consistency and transparency in reporting. For further information on Nature Portfolio policies, see our Editorial Policies and the Editorial Policy Checklist.

Statistics

For all statistical analyses, confirm that the following items are present in the figure legend, table legend, main text, or Methods section.

n/a	Confirmed	
☐	☒	The exact sample size (*n*) for each experimental group/condition, given as a discrete number and unit of measurement
☒	☐	A statement on whether measurements were taken from distinct samples or whether the same sample was measured repeatedly
☐	☒	The statistical test(s) used AND whether they are one- or two-sided<br>*Only common tests should be described solely by name; describe more complex techniques in the Methods section.*
☐	☒	A description of all covariates tested
☒	☐	A description of any assumptions or corrections, such as tests of normality and adjustment for multiple comparisons
☐	☒	A full description of the statistical parameters including central tendency (e.g. means) or other basic estimates (e.g. regression coefficient) AND variation (e.g. standard deviation) or associated estimates of uncertainty (e.g. confidence intervals)
☒	☐	For null hypothesis testing, the test statistic (e.g. *F*, *t*, *r*) with confidence intervals, effect sizes, degrees of freedom and *P* value noted<br>*Give P values as exact values whenever suitable.*
☒	☐	For Bayesian analysis, information on the choice of priors and Markov chain Monte Carlo settings
☒	☐	For hierarchical and complex designs, identification of the appropriate level for tests and full reporting of outcomes
☒	☐	Estimates of effect sizes (e.g. Cohen's *d*, Pearson's *r*), indicating how they were calculated

Our web collection on statistics for biologists contains articles on many of the points above.

Software and code

Policy information about availability of computer code

Data collection	N/A (Code was not involved in the creation of data for this study)
Data analysis	We conducted weighted prevalence/incidence estimation, transmission modeling, and meta-analyses using R software (version 4.5.2). For phylogenetic analyses, we created a temporally-resolved phylogeny using a modified version of the Nextstrain83 mpox workflow (https://github.com/nextstrain/mpox). We estimated reproduction number parameters via Markov chain Monte Carlo sampling (MCMC) using BEAST2 (version 2.7.8). Code for implementing the adapted version of the birth-death skyline model is available from https://github.com/nicfel/bdsky. We recorded structured phylogenetic trees from model output and simulated corresponding genetic sequences using Seq-Gen (version 1.3.4).

For manuscripts utilizing custom algorithms or software that are central to the research but not yet described in published literature, software must be made available to editors and reviewers. We strongly encourage code deposition in a community repository (e.g. GitHub). See the Nature Portfolio guidelines for submitting code & software for further information.

Data

Policy information about availability of data

All manuscripts must include a data availability statement. This statement should provide the following information, where applicable:

- Accession codes, unique identifiers, or web links for publicly available datasets
- A description of any restrictions on data availability
- For clinical datasets or third party data, please ensure that the statement adheres to our policy

Individual-level testing and clinical outcomes data reported in this study (Figures 1–2) are not publicly shared due to privacy protections for patient electronic health records. Individuals wishing to access disaggregated data, including data reported in this study, should submit requests for access to sara.y.tartof@kp.org. Requests will receive a response within 14 days. De-identified data (including, as applicable, participant data and relevant data dictionaries) will be shared upon approval of analysis proposals with signed data-access agreements in place. Source data not requiring information from patient electronic health records are provided for analyses presented in Figures 3–5.

Research involving human participants, their data, or biological material

Policy information about studies with human participants or human data. See also policy information about sex, gender (identity/presentation), and sexual orientation and race, ethnicity and racism.

Reporting on sex and gender	We restricted primary analyses of Kaiser Permanente Southern California patients to males, as ascertained from electronic health records. Our analyses include individuals who identify as transgender based on electronic health record data (N=268; 3.4%), although this total may not represent all cohort members with transgender or non-binary identity in the event that this identity was not disclosed to clinical providers.
Reporting on race, ethnicity, or other socially relevant groupings	We stratify the population according to race/ethnicity, as self-reported by individuals at enrollment in Kaiser Permanente Southern California health plans and ascertained from electronic health record data. Categories included White, non-Hispanic; Black, non-Hispanic; Asian/Pacific Islander, non-Hispanic; Hispanic of any race; and other or unknown race.
Population characteristics	Our study cohort comprised 7,930 males aged 16-52 years on May 1, 2024 who were enrolled in Kaiser Permanente Southern California healthcare plans, with ≥1 year of prior enrollment and ≥1 anorectal test for Chlamydia trachomatis or Neisseria gonorrhoeae infection (an indication reserved for men who have sex with men). Our age-based restrictions limited the population to individuals born after cessation of smallpox vaccination for the US general public, improving comparability of our estimates to those of other prospective testing studies and constraining the sample to age groups experiencing the greatest risk of mpox and other STIs. We restricted analyses to eligible males who had any appointment or received any laboratory test, prescription fill, immunization, or other care at the West Los Angeles Medical Center or Los Angeles Medical Center. As these were the KPSC facilities diagnosing the greatest number of mpox cases since 2022, individuals receiving care from these facilities were expected to have the greatest likelihood of mpox diagnosis in the event of clinical illness.
Recruitment	We did not conduct active recruitment of study participants. We analyzed data captured through patient electronic health records, and tested for MPXV in remnant anorectal specimens initially collected for N. gonorrhoeae/C. trachomatis testing in the context of routine care delivery.
Ethics oversight	The study was reviewed and approved by the Kaiser Permanente Southern California Institutional Review Board, with a waiver of the requirement for informed consent due to secondary use of electronic health records and clinical specimens. The study was conducted in compliance with all relevant ethical regulations.

Note that full information on the approval of the study protocol must also be provided in the manuscript.

Field-specific reporting

Please select the one below that is the best fit for your research. If you are not sure, read the appropriate sections before making your selection.

☐ Life sciences ☒ Behavioural & social sciences ☐ Ecological, evolutionary & environmental sciences

For a reference copy of the document with all sections, see nature.com/documents/nr-reporting-summary-flat.pdf

Behavioural & social sciences study design

All studies must disclose on these points even when the disclosure is negative.

Study description	Our primary analyses were quantitative and addressed electronic health records data. We conducted additional quantitative analyses of prevalence estimates from prior surveillance studies, and of MPXV sequence data.
Research sample	We aimed to study mpox and MPXV infections in men who have sex with men, who comprise a hidden population. Eligible individuals were males born between January 1, 1972 and May 1, 2008 who had been members of Kaiser Permanente Southern California health plans continuously since at least 1 May, 2023 (allowing enrollment gaps <45 days), ensuring capture of prior-year healthcare utilization and recent STI history. Our age-based restrictions limited the population to individuals born after cessation of smallpox

	vaccination for the US general public, improving comparability of our estimates to those of other prospective testing studies, and constraining the sample to age groups (16-52 years) experiencing the greatest risk of mpox and other STIs. We further restricted eligibility to individuals who: received ≥1 test anorectal N. gonorrhoeae/C. trachomatis test after 1 May, 2023, as such testing is indicated only for MSM, and who over the same period had any appointment or received any laboratory test, prescription fill, immunization, or other care at the West Los Angeles Medical Center or Los Angeles Medical Center. As these were the KPSC facilities diagnosing the greatest number of mpox cases since 2022, individuals receiving care from these facilities were expected to have the greatest likelihood of mpox diagnosis in the event of clinical illness. Data analyzed in this study comprise all healthcare interactions that KPSC members experience within the healthcare system (captured by electronic health records); out-of-network care is further captured by insurance claim reimbursements, enabling near-complete monitoring of healthcare delivery for the study population. The Kaiser Permanente Southern California membership broadly reflects the racial, ethnic, and demographic distribution of the surrounding regional population. We note, however, that MSM comprise a "hidden" population and it is not possible to verify whether the study population is representative of all MSM.
Sampling strategy	We included data from all individuals meeting eligibility criteria; as our primary analytic objective was descriptive (aimed at prevalence estimation), our study did not include a pre-determined minimum sample size chosen to support null hypothesis testing at a desired level of statistical power. Power analyses for the primary hypothesis test—that prevalence of PCR positivity in anorectal specimens was equal to the level expected when accounting for clinically-detected cases alone (0.035%)—revealed our sample size yielded >80% power at two-sided p<0.05 to reject the null hypothesis under scenarios where the true ratio of infections to reported cases was >10.3. Statistical power was expected to exceed 90% if this ratio exceeded 12.8 (Figure S9).
Data collection	Data were recorded within electronic health records at Kaiser Permanente Southern California (KPSC). The KPSC healthcare system provides comprehensive, integrated care to members; electronic health records (EHRs) record all diagnoses, clinical notes, laboratory tests, procedures, immunizations, and pharmacy prescription fills. Insurance reimbursement data capture out-of-network care, and history of vaccination with JYNNEOS (modified Vaccinia Ankara) is automatically reconciled with the California Immunization Registry, to which providers statewide are required to report all vaccine administrations. We supplemented routine mpox testing for clinical diagnosis with polymerase chain reaction (PCR) testing for MPXV in remnant anorectal specimens collected in the context of N. gonorrhoeae/C. trachomatis testing. We recorded results of PCR tests for MPXV-specific and non-Variola Orthopoxvirus probes, including qualitative (positive/negative) and quantitative (cycle threshold value) results. Researchers were not blinded to participant outcomes or exposures because the study used retrospectively collected information.
Timing	Our study period encompassed May 29 to November 13, 2024 for the primary analyses. For phylogenetic analyes, we included all MPXV sequences collected between May 1 ,2022 and December 31, 2024.
Data exclusions	No data were excluded from analyses.
Non-participation	N/A (no drop-out or non-participation as our study involved passive data collection only).
Randomization	Our study did not involve random allocation of individuals to exposure groups. For primary analyses, to address differences in characteristics of individuals from whom we received or did not receive a specimen for MPXV testing during the study period, we adjusted estimates of mpox incidence and MPXV infection prevalence within the KPSC study cohort via stabilized inverse propensity weighting. We computed stabilized inverse propensity-of-testing weights using a logistic regression model defining receipt of testing as the outcome; covariates included individuals' age group, race/ethnicity, health insurance payment source, neighborhood deprivation index (a community-level proxy for individual socioeconomic status), prior-year healthcare utilization across outpatient settings and history of emergency department presentation or inpatient admission, prior-year STI testing, receipt of HIV pre-exposure prophylaxis, receipt of doxycycline post-exposure prophylaxis, receipt of JYNNEOS, prior diagnoses of HIV, syphilis, gonorrhea, chlamydia, mpox, or other STIs, and prior diagnosis of alcohol or drug abuse.<br><br>For analyses addressing vaccine effectiveness, we estimated VE_D used a case-control framework comparing adjusted odds of prior vaccination among cases diagnosed with laboratory-confirmed mpox to controls diagnosed with laboratory-confirmed gonorrhea during the study period. This strategy was anticipated to mitigate confounding based on the expectation that receipt of JYNNEOS could be associated with individuals' risk of STI exposure as well as their engagement with sexual health services and likelihood of being diagnosed, if infected; whereas JYNNEOS would not be expected to alter individuals' risk of gonorrhea, gonorrhea cases were expected to resemble mpox cases in sexual risk characteristics and healthcare-seeking behavior. We defined the product θ_S θ_P as the adjusted odds ratio of prior JYNNEOS vaccination (receipt of any doses, 1 dose, or ≥2 doses) among mpox cases relative to controls diagnosed with gonorrhea, and estimated this term via conditional logistic regression. We defined matching strata on individuals' HIV infection status and (among HIV-negative individuals) receipt or non-receipt HIV PrEP; receipt of doxycycline post-exposure prophylaxis; and history of any syphilis diagnosis. Models further controlled for individuals' age group, receipt of N. gonorrhoeae/C. trachomatis testing in the prior year, and commercial or non-commercial insurance source (expected to proxy socioeconomic status) via covariate adjustment.

Reporting for specific materials, systems and methods

We require information from authors about some types of materials, experimental systems and methods used in many studies. Here, indicate whether each material, system or method listed is relevant to your study. If you are not sure if a list item applies to your research, read the appropriate section before selecting a response.

Materials & experimental systems

n/a	Involved in the study
☒	☐ Antibodies
☒	☐ Eukaryotic cell lines
☒	☐ Palaeontology and archaeology
☒	☐ Animals and other organisms
☐	☒ Clinical data
☒	☐ Dual use research of concern
☒	☐ Plants

Methods

n/a	Involved in the study
☒	☐ ChIP-seq
☒	☐ Flow cytometry
☒	☐ MRI-based neuroimaging

Clinical data

Policy information about clinical studies

All manuscripts should comply with the ICMJE guidelines for publication of clinical research and a completed CONSORT checklist must be included with all submissions.

Clinical trial registration: N/A (not a clinical trial)

Study protocol: N/A (this was a descriptive study addressing infection prevalence using passively-collected data from electronic health records and re-testing of remnant anorectal specimens; as such, the study did not have a pre-specified protocol)

Data collection: We monitored for mpox diagnoses in patients' electronic health records from May 29 to November 13, 2024, and tested anorectal secimens collected from eligible cohort members for MPXV over the same study period. We used all available MPXV clade IIb genomes found on Genbank through December 31, 2024.

Outcomes: The study outcomes included clinical diagnosis with laboratory-confirmed mpox, as ascertained from participants' electronic health records, and subclinical infection with MPXV, as ascertained from results of polymerase chain reaction testing of anorectal specimens.

Plants

Seed stocks: *Report on the source of all seed stocks or other plant material used. If applicable, state the seed stock centre and catalogue number. If plant specimens were collected from the field, describe the collection location, date and sampling procedures.*

Novel plant genotypes: *Describe the methods by which all novel plant genotypes were produced. This includes those generated by transgenic approaches, gene editing, chemical/radiation-based mutagenesis and hybridization. For transgenic lines, describe the transformation method, the number of independent lines analyzed and the generation upon which experiments were performed. For gene-edited lines, describe the editor used, the endogenous sequence targeted for editing, the targeting guide RNA sequence (if applicable) and how the editor was applied.*

Authentication: *Describe any authentication procedures for each seed stock used or novel genotype generated. Describe any experiments used to assess the effect of a mutation and, where applicable, how potential secondary effects (e.g. second site T-DNA insertions, mosiacism, off-target gene editing) were examined.*
